# From nutrient-based to food-based assessment: the evolution of inflammatory indices and their significance for metabolic syndrome and type 3 diabetes mellitus

**DOI:** 10.3389/fnut.2026.1818219

**Published:** 2026-05-14

**Authors:** Li Tao, Yangtao Liu, Jiayi Li, Jie Zhang, Huan Zhao, Kang Luo, Yuan Gao, Jun Mu, Qinghuan Yang, Zhiwen Yan

**Affiliations:** 1 Department of Clinical Nutrition, People’s Hospital of Chongqing Hechuan, Chongqing, China; 2 Department of Neurology, The First Affiliated Hospital of Chongqing Medical University, Chongqing, China; 3 Department of Geriatrics, The First Affiliated Hospital of Chongqing Medical University, Chongqing, China

**Keywords:** Alzheimer’s disease, dietary inflammatory index (DII), food inflammation index (FII), food matrix, gut-metabolism-brain axis, metabolic syndrome, precision nutrition, type 3 diabetes mellitus

## Abstract

Chronic low-grade inflammation has emerged as the pivotal driver connecting metabolic syndrome (MetS) and type 2 diabetes mellitus (T2DM) to neurodegenerative disorders, a pathological continuum increasingly recognized as “Type 3 Diabetes Mellitus” (T3DM). Diet, as a primary modifiable lifestyle factor, plays a dual role as both an inflammatory trigger and a potential therapeutic target. This review systematically delineates the methodological evolution of dietary inflammatory indices, shifting from the reductionist, nutrient-centric logic of the Dietary Inflammatory Index (DII) to the systemic, “food-matrix-based” logic of the recently proposed Food Inflammation Index (FII). We provide an in-depth mechanistic synthesis of the gut-metabolism-brain axis, illustrating how high-inflammatory diets initiate a malignant cascade: beginning with gut dysbiosis and barrier leakage, followed by immunometabolic reprogramming of adipose tissue, and culminating in the “Trojan Horse” effect at the blood–brain barrier. This process facilitates amyloid-beta accumulation and bioenergetic crises, forming the molecular basis of T3DM. While the DII remains an irreplaceable tool for large-scale historical and cross-cultural epidemiological research, we argue that the FII represents an important methodological advancement toward precision nutrition. By quantifying intra-group heterogeneity and capturing whole-food effects, the FII is designed to address the clinical “translation bottleneck” of nutrient-based assessments. Furthermore, we explore the clinical integration of the Food Inflammation Scores of Individuals (FISI) with digital health platforms and artificial intelligence, proposing novel, pre-emptive tools such as Children’s FISI (C-FISI) and Pregnancy FISI (P-FISI) for life-cycle management. This review bridges the gap between nutrition science and neuro-metabolic pathology, providing a novel theoretical framework and practical tools for the integrated management of MetS and the early prevention of T3DM.

## 1 Introduction

Globally, non-communicable diseases (NCDs) have emerged as the most formidable challenge to human health. Data from the World Health Organization (WHO) indicate that NCDs account for over 41 million deaths annually, representing 74% of all global mortality. Among these, cardiovascular diseases (CVD), cancer, chronic respiratory diseases, and diabetes are the primary causes of death (1). Of even greater concern is the increasingly early onset of NCDs, which severely undermines the health of the working-age population and hampers socio-economic development. Consequently, the effective prevention and control of NCDs have become critical imperatives in global public health.

Among the various NCDs, metabolic syndrome (MetS)—a clinical cluster characterized by abdominal obesity, dyslipidemia, hypertension, and hyperglycemia—has become the primary driver of the global epidemics of CVD and type 2 diabetes mellitus (T2DM) (2, 3). According to estimates from the International Diabetes Federation (IDF), the global prevalence of MetS is as high as 20–25%, and this figure continues to rise due to the Westernization of lifestyles and the intensification of population aging (4). MetS not only escalates the risk of T2DM and CVD but is also closely associated with multiple other NCDs, including non-alcoholic fatty liver disease (NAFLD) (5), chronic kidney disease (CKD) (6), polycystic ovary syndrome (PCOS) (7), and certain malignancies. Therefore, early identification and comprehensive intervention for MetS are of paramount importance for the prevention and management of associated NCDs.

In recent years, as research into the pathological mechanisms of MetS has deepened, chronic low-grade inflammation has been increasingly recognized as the core driver of its onset and progression (8). Growing evidence suggests that each component of MetS, including abdominal obesity, dyslipidemia, and hyperglycemia, is closely linked to elevated systemic inflammatory levels (9). For instance, obese adipose tissue secretes a plethora of pro-inflammatory cytokines, such as tumor necrosis factor-alpha (TNF-*α*) and interleukin-6 (IL-6). These cytokines not only induce insulin resistance but also damage vascular endothelial cells, thereby promoting the development of atherosclerosis (10, 11). Furthermore, hyperglycemia and dyslipidemia activate inflammatory responses through multiple pathways, including the formation of advanced glycation end-products (AGEs) and the induction of vascular endothelial dysfunction mediated by oxidized low-density lipoprotein (ox-LDL) (12). These substances activate immune cells and trigger the release of inflammatory mediators, further exacerbating metabolic disturbances and vascular injury.

Notably, chronic low-grade inflammation is not only tied to MetS and its related metabolic disorders but also shares a complex pathological link with neurodegenerative diseases, particularly Alzheimer’s disease (AD) (13). AD is a neurodegenerative disorder primarily characterized by progressive cognitive dysfunction and memory loss, and it remains the most prevalent form of senile dementia. Historically, the pathogenesis of AD was thought to center on the deposition of amyloid-beta (Aβ) plaques and the formation of neurofibrillary tangles (NFTs) (14). However, emerging research indicates that AD progression is also intimately associated with brain-specific insulin resistance and impaired glucose metabolism (15, 16). Based on these findings, de la Monte and Wands first proposed the concept of “Type 3 Diabetes Mellitus (T3DM)” in 2008, positing that AD is essentially a form of brain-specific insulin resistance. In this state, the responsiveness of cerebral neurons to insulin declines, leading to impaired glucose utilization, which ultimately triggers neuronal dysfunction and death (17). This paradigm is supported by a growing body of evidence, with Kellar and Craft further elucidating the central role of cerebral insulin resistance in AD pathogenesis and its potential therapeutic implications (18).

Although the precise mechanisms linking peripheral metabolic disturbances to cerebral insulin resistance remain to be fully elucidated, recent evidence identifies chronic low-grade systemic inflammation as the core driver connecting metabolic dysfunction with neurodegeneration. On one hand, T2DM and neurodegenerative diseases share common pathophysiological mechanisms, in which inflammation and gliotransmitters play multifaceted roles (19). Inflammatory cytokines can compromise the blood–brain barrier (BBB), directly damaging neurons and promoting Aβ deposition and NFTs formation (20). On the other hand, the inflammatory response exacerbates cerebral insulin resistance, further deteriorating neuronal energy metabolism and accelerating the neurodegenerative process (20). This inflammation-metabolism-neurodegeneration pathological axis is evident across various neurodegenerative conditions (21). Thus, controlling chronic low-grade inflammation and maintaining stable cerebral glucose metabolism are vital for preventing or delaying AD progression. Given these shared pathways, Michailidis et al. have systematically characterized AD as T3DM, emphasizing the overlapping pathophysiological signatures between AD and T2DM (22).

Among the numerous modifiable risk factors, diet has garnered significant attention due to its accessibility and feasibility for intervention. As a primary controllable lifestyle factor for modulating systemic inflammatory status, diet plays a pivotal role in the prevention of chronic diseases such as MetS, T2DM, and AD (23). In recent years, the Dietary Inflammatory Index (DII) has been extensively utilized in nutritional epidemiology to assess the inflammatory potential of the diet. Developed by Shivappa et al. in 2014, the DII scores various food items and nutrients based on their pro- or anti-inflammatory properties, providing a comprehensive reflection of an individual’s overall dietary inflammatory load (24). Extensive research has demonstrated that high DII scores are significantly associated with an increased risk of MetS (25–27), T2DM (28), and AD.

To overcome the inherent limitations of the DII, researchers have sought more streamlined, practical, and precise methodologies for assessing dietary inflammation. In 2024, Wang et al. introduced the Food Inflammation Index (FII) (29). Unlike the nutrient-based DII, the FII directly evaluates the inflammatory potential of various whole foods, offering a more intuitive assessment of their impact on inflammatory status. Compared with the DII, the FII is more readily understood and applied, facilitating healthier dietary choices for consumers. More importantly, the FII reveals key inflammatory components within foods and identifies intra-group heterogeneity, providing a novel tool for precision nutritional interventions (29). While the DII marked a significant advancement, its reliance on complex nutrient-based calculations has limited its widespread application in clinical practice and public health. The recently proposed FII addresses this by directly assessing food-specific inflammatory characteristics. However, a systematic comparison between these two methodologies and a comprehensive summary of their application in MetS and its organ-specific manifestations—particularly T3DM—remain absent from the current literature.

Consequently, this review aims to: (1) systematically delineate the methodological evolution from the DII to the FII and its scientific foundation; (2) provide an in-depth analysis of the multi-organ synergistic pathogenic mechanisms mediated by dietary inflammation, specifically focusing on how inflammation bridges metabolic dysfunction and cerebral insulin resistance; (3) comprehensively summarize the latest evidence regarding the DII and FII in the context of MetS, T2DM, and AD; and (4) prospect the application of the FII in precision nutritional intervention and the early prevention and control of chronic diseases. By integrating inflammatory assessment methods from the nutrient to the food level, this review establishes a novel theoretical framework and provides practical tools for the integrated management of MetS and the early prevention of T3DM.

## 2 Literature search strategy and selection criteria

To construct a comprehensive and rigorous narrative review, a systematic literature search was conducted across databases including PubMed, Web of Science, and Scopus, covering the period from database inception to March 2026. The search strategy utilized combinations of the following keywords and Medical Subject Headings (MeSH) terms: (“Dietary Inflammatory Index” OR “DII” OR “Food Inflammation Index” OR “FII” OR “Empirical Dietary Inflammatory Pattern” OR “EDIP”) AND (“Metabolic Syndrome” OR “Type 2 Diabetes” OR “Alzheimer’s Disease” OR “Type 3 Diabetes” OR “Cognitive Decline”).

Inclusion, Exclusion, and Screening Process: Initial screening was conducted by reviewing the titles and abstracts of retrieved articles to determine their relevance to the core topics (DII/FII, MetS, T2DM, and AD). Full texts of potentially relevant articles were then evaluated by the authors to confirm eligibility. We included peer-reviewed articles written in English, primarily focusing on large-scale observational studies (cross-sectional and prospective cohorts), recent meta-analyses, and robust mechanistic studies. Case reports, non-peer-reviewed preprints, and studies lacking validated dietary assessment methodologies were excluded.

Quality Assessment: Given the nature of a narrative review, formal quantitative quality scoring tools (e.g., Newcastle-Ottawa Scale) were not strictly applied. Instead, a qualitative quality assessment was conducted during the literature selection process. We prioritized studies with robust methodological designs, large sample sizes, and extensive follow-up periods. Furthermore, a critical appraisal approach was integrated into the evaluation of clinical evidence. We specifically assessed potential sources of methodological heterogeneity and study limitations (e.g., FFQ recall bias, residual confounding, and the risk of reverse causation in cognitive studies), which are explicitly discussed in the respective sections of this review.

## 3 The evolution of dietary inflammatory indices

### 3.1 Establishing the milestone: literature-based global standardized assessment

In the history of research concerning the relationship between diet and chronic inflammation, assessment methodologies have undergone a profound evolution—shifting from a reductionist nutrient-centric approach to systemic dietary pattern evaluations, and ultimately toward precision food-based logic. Early studies primarily focused on the biological activities of single nutrients, such as vitamins C and E or n-3 polyunsaturated fatty acids (30, 31). While these provided a robust mechanistic foundation, they struggled to account for the synergistic effects between components in complex diets and the holistic impact of whole-food matrices on a host’s inflammatory status.

To overcome these constraints, Hébert and colleagues initially developed and, in 2014, refined the landmark DII. According to the official review by the DII creators (32), the second-generation DII was derived from a meta-analysis of 1,943 original research articles, encompassing 45 dietary parameters—including flavonoids—and targeting six core inflammatory biomarkers (IL-1β, IL-6, IL-10, TNF-*α*, and C-reactive protein [CRP]) (24). A central innovation of the DII was the introduction of a global dietary reference database spanning 11 countries, which utilized Z-score standardization to resolve the challenges of incomparable measurements and disparate international dietary surveys. Hébert et al. emphasized that employing global norms, rather than study-specific means, effectively mitigated “intra-method correlated errors” often caused by a narrow range of exposure variability within a single population, thereby ensuring the universal applicability and comparability of the index across diverse cultural contexts (32).

### 3.2 Precision iterations: population adaptation and energy normalization

As the DII framework gained widespread application, its methodological logic expanded toward diversification and specialization. To address the potential interference of total energy intake on inflammatory scoring, researchers developed the Energy-Adjusted Dietary Inflammatory Index (E-DII). Hébert et al. explicitly identified two countervailing effects in dietary assessment: the “total intake effect,” where increased energy consumption leads to a synchronized rise in both pro- and anti-inflammatory nutrients, and the “healthy eater effect,” characterized by individuals intentionally choosing nutrient-dense but energy-sparse foods (32). These phenomena often produce negative correlation biases that complicate inflammatory profiling. The E-DII addresses these complexities by converting food parameters into nutrient densities (per 1,000 kcal), significantly enhancing its predictive power for metabolic-related chronic diseases.

Concurrently, to align with unique nutritional requirements across different life stages, the Children’s Dietary Inflammatory Index (C-DII) (33) and the Pregnancy Dietary Inflammatory Index (P-DII) were established. These versions optimized reference databases to match the physiological signatures of growth, development, and gestation. Furthermore, collaborative research has successfully achieved the cultural localization and validation of the DII within the context of the Chinese diet, confirming its cross-cultural robustness.

### 3.3 Logical shift: data-driven approaches and multidimensional lifestyle integration

To overcome potential publication biases inherent in traditional literature-based methods, the underlying logic of assessment tools began to shift toward a “data-driven” orientation. The Empirical Dietary Inflammatory Pattern (EDIP) (34), proposed by scholars at Harvard University, was derived directly from large-scale cohort studies. By utilizing clinical regression to select food groups most closely associated with circulating inflammatory markers, the EDIP enhanced the objectivity of predicting clinical outcomes (32).

However, Hébert et al. (2019) offered a critical reflection on data-driven indices, noting that they often suffer from “idiosyncrasies” of the particular populations from which they derive. For instance, dietary patterns identified in Western cohorts—which often include items like pizza or processed meats—may lack representativeness or relevance in rice-based cultures such as those in Asia. These limitations prompted researchers to re-evaluate the universality of assessment tools. Simultaneously, the Lifestyle Inflammation Score (LIS) (35) emerged to expand the scope of risk assessment beyond diet alone, integrating body mass index (BMI), tobacco exposure, and physical activity into a comprehensive metric for studying multi-factor synergistic pathogenic mechanisms.

### 3.4 Methodological shift: from molecular composition to the food matrix

**Figure 1:**
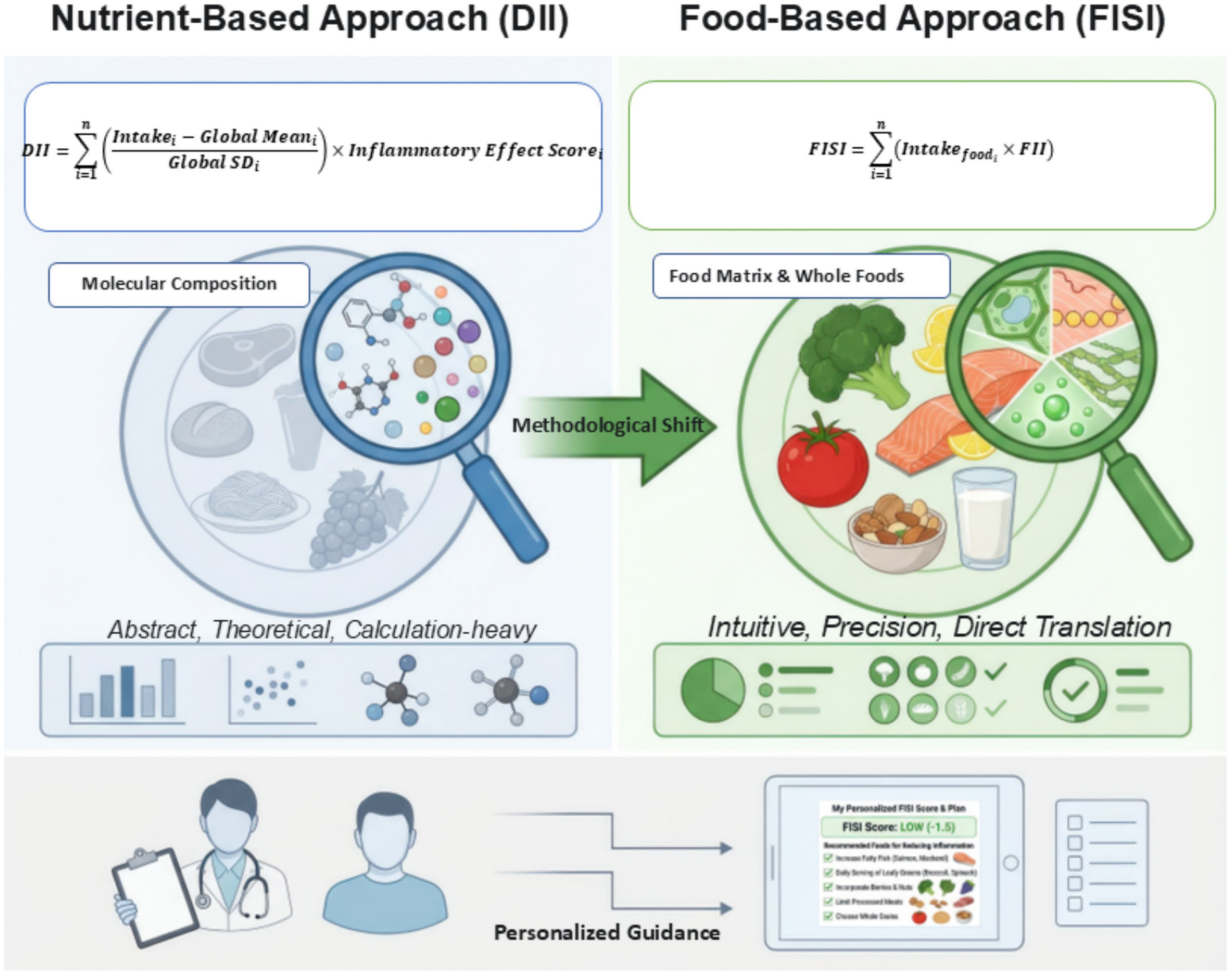
The methodological transition in dietary inflammation assessment: from the abstract DII to the clinically actionable FII (top left). The traditional DII relies on abstract nutrient calculations (molecular composition), which limits its intuitive application in clinical practice (top right). In contrast, the FII directly evaluates the inflammatory potential of whole food matrices, offering a more precise and easily translatable approach. (Bottom) FISI translates these FII values into personalized, evidence-based dietary guidance (e.g., specific food substitutions), directly empowering healthcare professionals and consumers. Note: DII, Dietary Inflammatory Index; FII, Food Inflammation Index; FISI, Food Inflammation Scores of Individuals; NRV, Nutrient Reference Value.

Dietary inflammation assessment is currently undergoing a profound conceptual shift from abstract molecular composition toward the “whole-food matrix effect.” As illustrated in Figure 1, traditional assessment logic (such as the DII) focuses on abstract chemical constituents. Despite the significant academic success of the DII, Hébert et al. (2019) candidly acknowledged in their summary that the “computation of DII scores can become quite nuanced and complicated,” a factor that has constrained its direct translation into clinical practice and public health settings. Clinicians often encounter a bottleneck characterized by a “knowledge of nutrients but ignorance of foods.”

With the rise of precision nutrition, the focus has shifted toward the complex food matrix and “whole-food effects.” FII, proposed by Wang et al. (29), is the first to quantitatively acknowledge and integrate the “food matrix effect.” By introducing weighting based on physiologically meaningful Nutrient Reference Values (NRVs), the FII bypasses the cumbersome Z-score calculation logic and directly evaluates the inflammatory potential of specific food items (e.g., distinguishing between different types of oils or vegetables). Such a shift is underscored by research into whole-food patterns like plant-based diets, which demonstrate that health benefits arise from synergistic biochemical interactions within the food matrix rather than isolated components (36). This transition from abstract calculation to an intuitive, precise, and directly translatable “food-based approach” not only addresses intra-group heterogeneity but also inaugurates a new era of clinical precision nutritional intervention through the Food Inflammation Scores of Individuals (FISI) (Table 1).

**Table 1:** Chronological evolution and methodological characteristics of dietary inflammatory indices.

Evolutionary stage	Core indices	Methodological basis	Standardization and normalization	Analytical granularity	Main advantages	Potential limitations	References
Nutrient-Centric Era	Single Nutrients (e.g., Vit C, n-3)	Biological assays & clinical observation	Non-standardized	Molecular/Nutrient level	Established foundational mechanistic evidence	Fails to account for food matrix synergies	(30, 31)
Global Pattern Era (2014)	DII	Literature-derived scoring (45 parameters)	Global Z-scores (Referent Database)	Holistic dietary patterns	First standardized quantitative scale; captures synergistic effects	Low interpretability for specific food-item selection	(24)
Refinement Era	E-DII, C-DII, P-DII, CHINA-DII	Population-specific literature review	Energy-adjustment; Specific referent DBs	Targeted demographics	Enhanced precision for specific life stages (e.g., pregnancy)	Primarily dependent on nutrient-level exposure	(33, 158–160)
Data-Driven Era	DIS/LIS, EDIP, EDIP-A,	Empirical derivation via biomarkers	Biomarker-based risk scoring	Food groups & Lifestyle factors	Reduced publication bias; assesses total inflammatory load	Limited external validity; lacks food-item precision	(34, 35, 79)
Precision Food Era (2024)	FII	Physiologically-based nutrient profiling	NRV weighting	Individual Food Items	Reveals intra-group heterogeneity; enables precise food choices within same food groups.	Dependent on the comprehensiveness of Food Composition Tables (FCTs).	(29)

DII, Dietary Inflammatory Index; E-DII, Energy-adjusted DII; C-DII, Children’s DII; P-DII, Pregnancy DII; CHINA-DII, Chinese Dietary Inflammatory Index; EDIP, Empirical Dietary Inflammatory Pattern; EDIP-A, Empirical Dietary Inflammatory Pattern-Asian; DIS, Dietary Inflammation Score; LIS, Lifestyle Inflammation Score; FII, Food Inflammation Index; NRV, Nutrient Reference Value; FCT, Food Composition Tables.

## 4 Biological mechanisms mediated by dietary inflammation

Dietary inflammatory potential is not an isolated biochemical state but rather a systemic catalyst driving interactive failure across multiple organs (37). Diets characterized by high inflammatory potential, as assessed by indices such as the DII and FII, are hypothesized to initiate a malignant cascade along the gut-metabolism-brain axis, constructing a pathological pathway that evolves from MetS to type 2 diabetes mellitus (T2DM) and ultimately to T3DM (38). This complex pathogenic process begins with the imbalance of gut microecology and the loss of barrier function, which is subsequently amplified by the immunometabolic reprogramming of adipose tissue. The resulting cytokine storm directly disrupts systemic and central insulin signaling pathways and eventually compromises the BBB, leading to a neurodegenerative endgame characterized by bioenergetic crises and pathological protein aggregation.

### 4.1 Origin: gut dysbiosis and pro-inflammatory leakage

The gastrointestinal tract serves as the primary line of defense against dietary antigens and acts as the pacemaker for systemic inflammatory responses (39). Chronic exposure to high-FII diets—particularly patterns rich in saturated fats and refined sugars but deficient in dietary fiber—disrupts microecological homeostasis and physical barrier integrity, thereby triggering the systemic dissemination of gut-derived inflammation (40).

High-inflammatory diets exert significant selective pressure on gut microbiota, leading to microbial drift. Specifically, high-fat, low-fiber diets deprive key probiotics, such as *Bifidobacterium* and *Akkermansia muciniphila*, of their metabolic substrates. This results in diminished production of short-chain fatty acids (SCFAs) and forces bacteria to erode the host’s intestinal mucus layer, weakening the primary biological barrier (41). This microenvironmental shift induces the overproliferation of Gram-negative bacteria, such as *Enterobacteriaceae*. A direct consequence of dysbiosis is the disintegration of the intestinal epithelial physical barrier. Under normal conditions, tight junction proteins, including Occludin, Claudin-5, and Zonula Occludens-1, maintain a rigorous defense line (42). However, local oxidative stress induced by pro-inflammatory diets downregulates the expression of these proteins, increasing intestinal permeability—a phenomenon known as “leaky gut” (43). The compromised barrier allows lipopolysaccharides (LPS) accumulated in the intestinal lumen to translocate into the systemic circulation. LPS binds to the TLR4 and CD14 complexes on the surface of bone marrow-derived immune cells, activating the NF-κB signaling pathway and inducing metabolic endotoxemia (39). This represents the starting point of chronic low-grade inflammation and a key early driver of insulin resistance (IR) (44, 45).

Beyond the classical LPS pathway, emerging evidence reveals a more covert and lethal gut-brain mechanism: the cross-seeding of functional bacterial amyloids (FUBA) (46). Certain gut commensals in high-FII environments secrete amyloid fibers that share high structural homology with cerebral amyloid-beta (Aβ). This molecular mimicry not only persistently activates the immune system via Toll-like receptors but may also undergo retrograde transport via the vagus nerve or enter the central nervous system (CNS) through blood circulation when the intestinal barrier is damaged. This physical pathway enables misfolded proteins produced in the gut to act as “seeds,” inducing conformational changes, nucleation, and deposition of endogenous proteins within neurons, thus directly linking unhealthy diets to neurodegenerative pathology (47).

Crucially, this gut-driven inflammatory cascade is no longer confined to preclinical models. Recent high-impact clinical evidence from the Multiethnic Cohort–Adiposity Phenotype Study provided strong clinical support for this pathway in humans. Through 16S rRNA sequencing and MRI-derived adiposity phenotyping, Lozano et al. demonstrated that the pro-inflammatory potential of the diet (E-DII) increases visceral adipose tissue and total fat mass indirectly through specific gut microbial alterations (e.g., increased abundance of pathogenic *Fusobacteria* and *Escherichia-Shigella*) and elevated circulating lipopolysaccharide-binding protein (LBP) (48). Similarly, Liu et al. identified that stratified dietary inflammatory potential directly drives oral-gut microbiota discrepancies (such as the depletion of *Holdemanella*), which strongly correlate with multidomain cognitive decline in older adults (49). These human-derived multi-omics data provide compelling evidence supporting the concept that diet-induced gut dysbiosis and LPS translocation are the primary biological pacemakers for downstream metabolic-neurodegenerative pathologies.

### 4.2 Amplification: immunometabolic reprogramming of adipose tissue

In the pathological chain mediated by dietary inflammation, adipose tissue acts not merely as a passive reservoir for excess energy but as an inflammatory amplifier that converts local microenvironmental disturbances into systemic signals (50). The persistent caloric surplus from high-FII diets forces adipocytes into pathological hypertrophy. When the rate of cellular expansion outpaces angiogenic capacity, a local hypoxic microenvironment forms within the adipose tissue. This hypoxia triggers the stabilization and nuclear translocation of hypoxia-inducible factor-1alpha (HIF-1α), which initiates a pro-inflammatory gene transcription program, leading to the massive release of chemokines such as monocyte chemoattractant protein-1 (MCP-1) (51). This signaling cascade recruits circulating monocytes to infiltrate the adipose tissue, where they undergo a phenotypic switch—polarizing from anti-inflammatory M2 macrophages to pro-inflammatory M1 macrophages—under the stimulus of pro-inflammatory nutrients like palmitic acid. These M1 cells surround necrotic adipocytes to form characteristic “crown-like structures,” serving as “inflammatory factories” that persistently secrete TNF-*α*, IL-6, and IL-1β (50).

Concurrent with immune cell infiltration is the comprehensive dysfunction of adipose endocrine activity. In a pro-inflammatory state, the secretion of adiponectin, which possesses insulin-sensitizing and vasoprotective properties, is significantly inhibited, while levels of pro-inflammatory leptin and resistin undergo compensatory elevation. This adipokine imbalance not only exacerbates the clinical manifestations of MetS but also induces systemic vascular inflammation through the activation of the JAK/STAT pathway (52). Crucially, when the storage capacity of subcutaneous adipose tissue reaches its limit, an influx of free fatty acids (FFAs) overflows into the bloodstream. These saturated fatty acids act as endogenous ligands, binding to TLR4 on cells across multiple organs and further activating the NF-κB pathway, establishing a self-amplifying feed-forward inflammatory loop that paves the way for IR (51).

### 4.3 Mediation: molecular roadblocks in the insulin signaling pathway

Systemic IR serves as the central hub connecting MetS to neurodegenerative diseases, with diet-induced inflammatory mediators acting as “molecular interceptors (53).” Under physiological conditions, insulin binding to its receptor induces tyrosine phosphorylation of insulin receptor substrate-1 (IRS-1), activating the downstream PI3K/Akt pathway to facilitate glucose uptake (54). However, the high levels of circulating cytokines (e.g., TNF-*α*) and FFAs generated by high-FII diets ectopically activate intracellular stress-sensitive kinases, particularly c-Jun N-terminal kinase (JNK) and IκB kinase beta (IKKβ) (55, 56). These activated inflammatory kinases act retroactively on upstream signaling nodes, inducing abnormal serine phosphorylation of IRS-1 at specific sites, such as Ser307 (57, 58).

This erroneous serine phosphorylation causes conformational changes in the insulin receptor substrate, creating a steric hindrance effect that functions as a “molecular roadblock,” obstructing the normal coupling between the insulin receptor and its substrate and inhibiting downstream signal transduction. Consequently, Akt phosphorylation is impaired and the translocation of glucose transporter-4 (GLUT4) to the cell membrane fails, leading to a sharp decline in glucose uptake by skeletal muscle and adipose tissue (59). To maintain glycemic homeostasis, pancreatic beta cells are forced to compensatorily secrete more insulin, resulting in peripheral hyperinsulinemia. Paradoxically, this high peripheral insulin concentration downregulates the expression of insulin transporters on the BBB via a negative feedback mechanism, leading to a state of peripheral hyperinsulinemia coexisting with central hypoinsulinemia. This process not only establishes the pathology of T2DM but also leaves the cerebral parenchyma in a state of chronic insulin signal deprivation, forming the critical molecular basis for the onset of T3DM (60).

### 4.4 Invasion: deconstruction of the BBB and the trojan horse effect

The BBB is the vital physical and biological defense line maintaining the stability of the cerebral microenvironment. However, under prolonged dietary inflammatory attack, this barrier regresses from a protective shield to a funnel for toxin infiltration. Continuously elevated circulating pro-inflammatory factors, especially TNF-*α* and IL-1β, induce brain microvascular endothelial cells to overexpress matrix metalloproteinases, specifically MMP-9 (61). Activated MMP-9 specifically degrades tight junction proteins, such as Claudin-5 and Occludin, leading to the loss of physical integrity and a significant increase in BBB permeability. This structural loosening allows peripheral immune cells, bacterial endotoxins, and circulating inflammatory mediators to bypass the barrier and infiltrate the brain parenchyma, completing the transition of inflammatory signals from chemical transmission outside the CNS to a physical invasion within it.

Beyond physical disruption, dietary inflammation leads to a pathological remodeling of the transporter receptor profile on the BBB, creating a “Trojan Horse effect.” In a normal physiological state, low-density lipoprotein receptor-related protein 1 (LRP1) is responsible for transporting Aβ produced in the brain to the blood for clearance, while receptor for advanced glycation end-products (RAGE) mediates the influx of peripheral Aβ into the brain (62). However, the systemic oxidative stress environment induced by high-FII diets reverses this balance: it downregulates LRP1 expression while significantly upregulating RAGE levels (63). This reversal of transport polarity not only hinders the clearance of cerebral metabolic waste but also actively pumps peripherally produced Aβ and glycation products into the brain. Consequently, the brain transforms from an immune-privileged organ into a reservoir for systemic metabolic waste, setting the environmental stage for neurodegeneration.

### 4.5 Finale: brain energy crisis and the pathological evolution of T3DM

The accumulation of diet-induced inflammatory mediators and metabolic toxins within the brain triggers the pathological cascade of T3DM, centered on brain-specific IR. This stage is most prominently characterized by a bioenergetic crisis in neurons. Due to impaired BBB insulin transport and the desensitization of cerebral insulin signaling pathways, neurons are unable to effectively uptake and utilize glucose, falling into a state of severe energy deprivation (64). To maintain survival, mitochondria are forced into overwork, leading to electron transport chain uncoupling and the generation of massive amounts of reactive oxygen species (ROS). This oxidative stress further damages mitochondrial DNA, creating a vicious cycle of energy metabolism failure (17).

**Figure 2:**
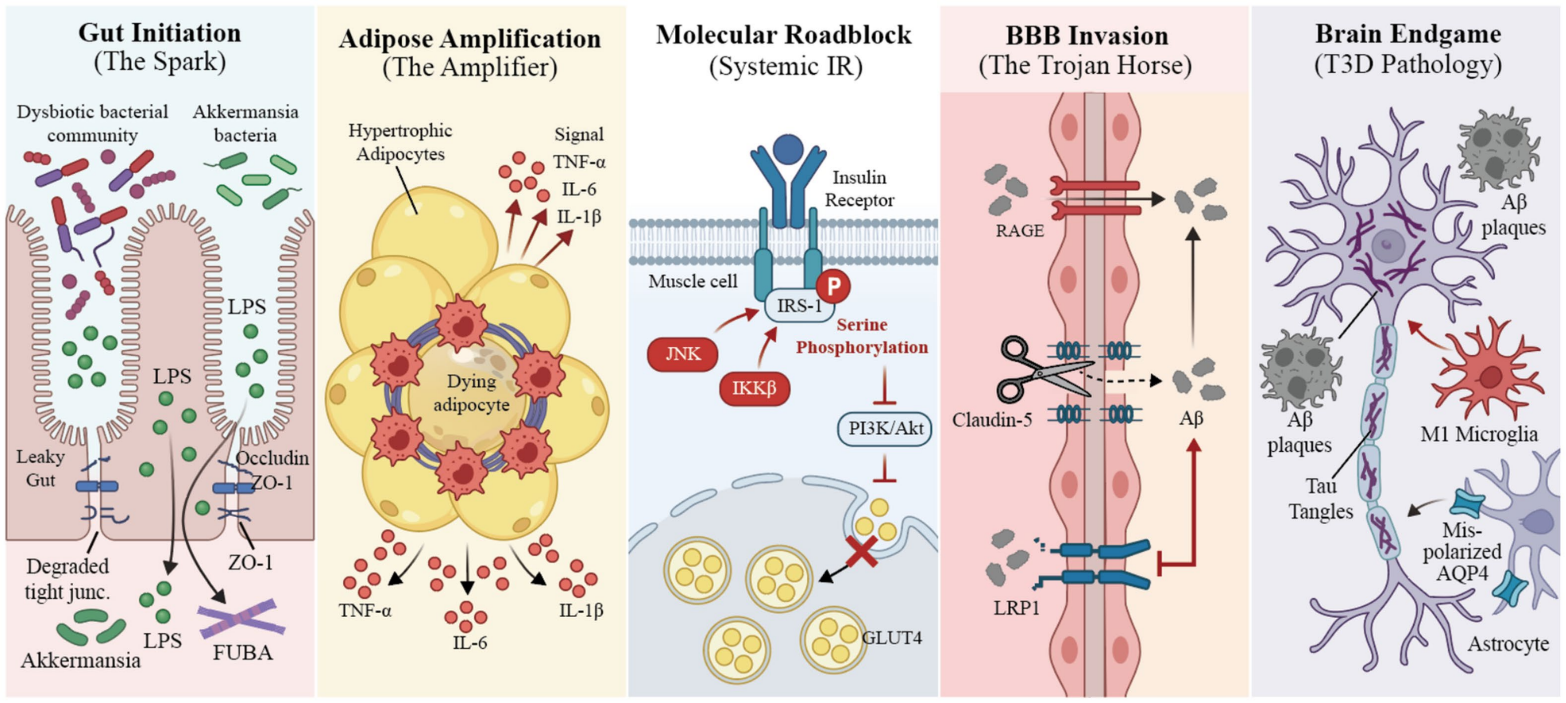
The multi-organ synergistic cascade of dietary inflammation along the gut-metabolism-brain axis. LPS, lipopolysaccharide; ZO-1, Zonula Occludens-1; FUBA, functional bacterial amyloids; TNF-*α*, tumor necrosis factor-alpha; IL-6, interleukin-6; IL-1β, interleukin-1 beta; IRS-1, insulin receptor substrate-1; JNK, c-Jun N-terminal kinase; IKKβ, IκB kinase beta; GLUT4, glucose transporter-4; BBB, blood–brain barrier; RAGE, receptor for AGEs; LRP1, low-density lipoprotein receptor-related protein 1; Aβ, amyloid-beta; AQP4, aquaporin-4; T3DM, type 3 diabetes mellitus.

At the molecular signaling level, the blockade of cerebral insulin signaling leads to the inactivation of downstream Akt kinase, thereby relieving the inhibition of glycogen synthase kinase-3beta (GSK-3β). Overactive GSK-3β acts as a molecular driver of neurodegeneration, directly catalyzing the hyperphosphorylation of Tau protein, which leads to the collapse of the microtubule cytoskeleton and the formation of neurofibrillary tangles—a hallmark pathological change in AD (65). Concurrently, microglia, the immune sentinels of the brain, undergo chronic activation under prolonged inflammatory stimulation. They not only lose their phagocytic capacity for clearing Aβ plaques but also release cytotoxic substances and excessively prune synapses, leading to the disconnection of neural networks. Most severely, inflammation disrupts the polar distribution of aquaporin-4 in astrocytes, paralyzing the glymphatic system and significantly reducing the brain’s ability to clear metabolic waste during sleep (66). Ultimately, the sparks of inflammation originating in the gut are amplified by the metabolic system and culminate in irreversible energy failure and protein deposition within the CNS, completing the pathological evolution from poor diet to dementia (Figure 2).

The pathological progression initiates with high-FII diet-induced gut dysbiosis and barrier leakage (Gut Initiation), triggering systemic dissemination of pro-inflammatory mediators (LPS and FUBA). These signals are amplified through adipose tissue immunometabolic reprogramming (Adipose Amplification), leading to systemic insulin resistance via stress-kinase-mediated IRS-1 serine phosphorylation (Molecular Roadblock). The cascade culminates in BBB disruption and a “Trojan Horse” effect, facilitating cerebral Aβ accumulation and neurodegenerative pathologies, including Tau aggregation, microglial activation, and glymphatic dysfunction (Brain Endgame).

## 5 Clinical research progress

### 5.1 DII and MetS

MetS has emerged as a primary driver of global epidemics of CVD and T2DM (1, 12, 13, 67). As a critical modifiable factor for modulating systemic inflammatory levels, diet has garnered substantial attention in the prevention and management of MetS. The DII and its refined variants have been extensively applied in clinical epidemiological research across diverse global populations.

### 5.2 Associations between DII/EDIP and MetS risk in general adult populations

The majority of evidence linking the DII to MetS stems from large-scale cross-sectional observational studies, highlighting significant racial and geographic variations. Multiple studies based on the National Health and Nutrition Examination Survey (NHANES) provide robust evidence that increasing DII scores are associated with a linear or non-linear upward trend in the prevalence of MetS and its core components, including abdominal obesity, hypertension, hyperglycemia, and hypertriglyceridemia (68). In middle-aged and older U. S. adults, a high DII is not only tied to higher overall MetS prevalence but is also specifically associated with reduced high-density lipoprotein cholesterol (HDL-C) and elevated fasting plasma glucose levels (15). This aligns with evidence identifying specific lipid biomarkers, such as palmitoleic acid, as significant indicators of metabolic risk in obese populations, highlighting the importance of granular lipid profiling in assessing diet-related metabolic disturbances (69). Conversely, research in certain Chinese urban populations found no significant overall association between the DII and MetS prevalence, though a high DII was correlated with elevated CRP levels among existing MetS patients (70). European studies present diverse outcomes: research in France and Croatia supports a positive association between the DII and both MetS and hypertension (71, 72), while the ORISCAV-LUX study in Luxembourg suggested that the DII primarily correlates with specific dyslipidemias, such as hypertriglyceridemia or low HDL-C, rather than the complete MetS cluster (73).

Prospective cohort studies further strengthen these findings. For instance, a 13-year follow-up of the French SU. VI. MAX cohort revealed that high DII scores significantly increased the incident risk of MetS (OR = 1.39), spanning dimensions of blood pressure, lipids, and glucose (72). In Asian populations, a 5-year follow-up of the Iranian Fasa cohort showed that E-DII scores increased the risk of new-onset MetS by 29% (74). Similarly, the Ravansar Non-Communicable Disease (RaNCD) cohort and the Tehran Lipid and Glucose Study found that high DII/EDIP scores were significantly associated with MetS risk, particularly regarding abdominal obesity and dyslipidemia (75–77). Furthermore, the Korean Genome and Epidemiology Study (KoGES_HEXA) demonstrated that high DII scores significantly increased MetS risk and its five components in women, especially postmenopausal individuals, with similar associations observed for abdominal obesity and hypertension in men (78). In Singapore, a multi-ethnic cohort confirmed the efficacy of the EDIP in predicting MetS risk (79). Beyond incidence, longitudinal data suggest a dynamic link between dietary inflammatory potential and disease severity; the PREDIMED-Plus trial demonstrated that transitioning to an anti-inflammatory diet synchronized with reductions in MetS severity scores, underscoring the feasibility of dietary intervention in reversing metabolic disturbances (80).

### 5.3 Critical appraisal of current observational evidence

While a substantial body of evidence links high DII/EDIP to MetS, these observational findings must be interpreted with methodological caution. As highlighted by a rigorous umbrella review (81), a significant proportion of DII-related meta-analyses exhibit high statistical heterogeneity ( $I^{2}$ > 50%) and potential small-study effects. A primary methodological flaw across these cohorts is the heavy reliance on self-reported Food Frequency Questionnaires (FFQs). Recent validations indicate that FFQs introduce considerable recall bias and social desirability bias compared to multiple 24-h dietary recalls (82). Furthermore, an authoritative scoping review by El-Khoury et al. (83) highlighted that due to limitations in regional Food Composition Tables, many primary studies arbitrarily omit critical anti-inflammatory components when calculating the DII, creating substantial inter-study heterogeneity. Consequently, the presence of residual confounding—such as unmeasured socio-economic disparities and physical frailty—suggests that current DII-MetS associations, though statistically robust, may overestimate the true causal effect size.

### 5.4 MetS in pregnant women and children

Gestation represents a critical window for the developmental origins of health and disease, where DII-related research has revealed significant intergenerational impacts. In women with a history of gestational diabetes mellitus (GDM), high DII diets during and after pregnancy markedly increase the risk of developing future T2DM, with obesity serving as a pivotal mediator (16). Crucially, a maternal pro-inflammatory diet may compromise the long-term health of offspring by altering breast milk composition (e.g., increasing pro-inflammatory fatty acid ratios) or the intrauterine immune environment, thereby increasing the risk of childhood type 1 diabetes (84, 85). A longitudinal study in Mexico further confirmed that cumulative high DII exposure from infancy to early adulthood is positively associated with higher MetS risk scores in later life, highlighting the enduring benefits of early-life dietary intervention (86). Concurrently, research in children and adolescents (C-DII/DII) indicates that high-inflammatory diets are closely linked to obesity, hypertension, and early metabolic abnormalities. A study in Ecuadorian children aged 6–12 showed that each unit increase in the DII was associated with a 1.20-fold increase in MetS risk (87). Systematic reviews and empirical studies across the U.S., Turkey, and Iran consistently identify high DII scores as independent risk factors for childhood obesity and adverse body composition (88, 89). In Turkish adolescents, C-DII scores were positively correlated with cardiometabolic risk indicators and inflammatory markers such as IL-6, foreshadowing increased cardiovascular risk in adulthood (90, 91).

### 5.5 Complications, specific populations, and long-term prognosis

As MetS progresses, the association between the DII and severe metabolic complications becomes increasingly apparent. In metabolic dysfunction-associated steatotic liver disease (MASLD/NAFLD), high DII/EDIP scores are independently associated not only with increased prevalence but also with disease severity, including liver fibrosis and hospitalization rates, supporting a “diet-inflammation-liver damage” pathological pathway (92, 93). In the context of CKD, pro-inflammatory diets are linked to declining renal function and increased CKD prevalence, particularly during the early stages of cardiovascular-kidney-metabolic (CKM) syndrome (94, 95). Furthermore, research in high-stress occupations (e.g., police officers, firefighters) suggests that occupational stress and pro-inflammatory diets may exert synergistic effects, exacerbating metabolic disturbances and inflammatory load (96, 97). Regarding prognosis, the DII possesses significant predictive value for survival outcomes in MetS patients. Multiple cohort studies consistently show that in middle-aged and older adults with MetS, high DII scores significantly increase the risk of all-cause and cardiovascular mortality (98, 99). These findings suggest that controlling dietary inflammation is a key strategy not only for preventing MetS but also for enhancing long-term quality of life.

### 5.6 DII and type 2 diabetes

DII and T2DM. As a chronic metabolic disorder, T2DM represents a primary risk factor for complications such as MetS, CVD, and nephropathy (100–102).

#### 5.6.1 Associations between DII/E-DII and T2DM risk in general adult populations

Cross-sectional analyses demonstrate a positive correlation between high DII scores and T2DM prevalence. Specifically, research in women found that those in the highest DII tertile had a 56% increased likelihood of T2DM, with this association being more pronounced in non-obese and low-income populations (103). After adjusting for lifestyle factors, the DII remained positively associated with T2DM, prediabetes, and insulin resistance (measured by homeostatic model assessment for insulin resistance, HOMA-IR) (104). These associations exhibit both consistency and specificity across regions and ethnicities. In Asia, baseline data from the Iranian RaNCD cohort showed that high DII scores increased T2DM prevalence by 48–61% (105, 106). Similarly, multi-ethnic research in Xinjiang, China, revealed that individuals in the high DII group were 3.27 times more likely to have T2DM compared to the lowest group (107). However, some studies, such as the Iranian TLGS, found no significant association with fasting glucose or overall T2DM prevalence after full adjustment, potentially due to differences in dietary assessment tools or sample characteristics (108).

In prospective cohort studies, a long-term follow-up of over 170,000 participants in the UK Biobank showed that high E-DII scores were independently associated with an 18% increased risk of incident T2DM, a risk further amplified by high salt intake (109). A dual-cohort study using UK Biobank and NHANES data confirmed that a lower E-DII was significantly associated with reduced T2DM incidence (110). Additionally, the E-DII framework has been linked to a higher risk of severe fatty liver disease, which often co-occurs with T2DM (92). Longitudinal data from the French E3N cohort and the China Health and Nutrition Survey (1997–2015) further support the causal inference that long-term pro-inflammatory diets lead to T2DM onset, with gender and BMI acting as significant modifiers (28, 111).

#### 5.6.2 Empirical dietary inflammatory pattern (EDIP) and T2DM

The EDIP provides a novel perspective by using data-driven methods to link specific food groups with inflammatory biomarkers. Research from the Women’s Health Initiative (WHI) found that the EDIP and the Empirical Dietary Index for Hyperinsulinemia (EDIH) had similar efficacy in predicting T2DM risk, suggesting that diet-induced inflammation and hyperinsulinemia may jointly drive T2DM development (112). In the Coronary Artery Calcification in Type 1 Diabetes (CACTI) study, higher EDIP scores were associated with increased MetS risk in both type 1 diabetes mellitus (T1DM) patients and controls, indicating the harm of pro-inflammatory patterns across different metabolic backgrounds (113). Furthermore, some studies have examined the DII alongside the Dietary Insulin Index (DIL). Notably, while high insulin indices typically correlate with T2DM risk, an intuitive negative correlation was observed in certain non-obese populations, suggesting that dietary indices may be complexly modified by factors like BMI (114, 115).

#### 5.6.3 T2DM in pregnant women and children

PDII research emphasizes specific metabolic complications during pregnancy and their intergenerational impact on T2DM risk. In women with a history of GDM, pro-inflammatory diets during and after pregnancy significantly increase the risk of future T2DM, with obesity as a key mediator (116). Another study found that even in women with lower overall DII scores, a high DII remains an independent predictor of progression to prediabetes (117). Importantly, the Danish National Birth Cohort (DNBC) revealed that high maternal EDIP scores during pregnancy were associated with a 16% increased risk of childhood T1DM in offspring, suggesting that maternal inflammation may influence long-term metabolic health via intrauterine immune programming (84). This risk is compounded by observations that pediatric patients with preexisting T1DM often exhibit systemic deficiencies in anti-inflammatory n-3 polyunsaturated fatty acids, potentially exacerbating the underlying inflammatory state (118). Concurrently, maternal smoking and the DII exhibit interactive effects on offspring T2DM risk, where an anti-inflammatory diet may mitigate the adverse impacts of smoking (119). Furthermore, breast milk from obese mothers often exhibits pro-inflammatory fatty acid profiles correlated with maternal DII scores, potentially forming a pathway for early metabolic risk transmission (85). Such findings underscore the critical role of maternal metabolic status, as seen in pregnancies complicated by diabetes, where dysregulated fatty acid supply can significantly impact early-life nutritional programming (120). In adolescents, C-DII research links high-inflammatory diets to impaired glucose tolerance (OR = 3.5) and dyslipidemia, foreshadowing future diabetes risk (90).

#### 5.6.4 Complications, comorbidities, and integrated management

As T2DM progresses, the DII’s association with severe complications becomes clearer. Regarding microvascular complications, high DII scores are significantly correlated with CKD prevalence, with risk increases of up to 67% (95). Patients with diabetic foot ulcers also exhibit higher DII scores, identifying the DII as an independent risk factor for ulcer occurrence and severity (121). Additionally, a high DII is associated with increased risk of diabetic sensorimotor polyneuropathy (DSPN) (75). In terms of macrovascular and metabolic comorbidities, pro-inflammatory diets are closely linked to increased risks of MASLD/NAFLD and fibrosis progression (122, 123). Furthermore, high DII scores significantly increase the risks of sarcopenia, mild cognitive impairment (MCI), and depression in T2DM patients, with BMI and systemic inflammatory markers playing critical mediating roles (124–126).

### 5.7 DII and T3DM

T3DM is a term used to describe the pathological signatures of AD, including cerebral insulin resistance, impaired glucose metabolism, and abnormal insulin-like growth factor signaling. Chronic low-grade systemic inflammation is the core driver connecting metabolic disturbances with neurodegeneration, making diet—the primary controllable modulator of inflammatory status—increasingly important in the prevention of T3DM and cognitive decline.

#### 5.7.1 Cross-sectional associations with cognitive function in middle-aged and older adults

Large-scale cross-sectional surveys of community-dwelling older adults reveal a consistent negative correlation between dietary inflammatory potential and multi-domain cognitive performance. Multiple studies from NHANES (2011–2014) provide strong evidence that high DII scores in adults aged 60 + are associated with lower scores in the Digit Symbol Substitution Test (DSST, assessing processing speed and executive function), Animal Fluency (AF, assessing language), and word delayed recall tests (127). After adjusting for confounders, high DII scores are significantly associated with a higher risk of cognitive impairment; specifically, individuals in the highest quartile face a 4.30-fold higher risk in the DSST compared to the lowest group (128). When the DII exceeds a threshold of 3.0, the risk of cognitive decline escalates sharply, following a non-linear dose–response relationship (129).

These associations have also been validated in Asian populations. Data from the 2018 China Health and Nutrition Survey (CHNS) indicate that high DII scores are significantly associated with increased risk of cognitive impairment (130). Research on overweight and obese Chinese residents further suggests that metabolic load may exacerbate the adverse effects of inflammatory diets on cognition (131). Another detailed assessment found that the E-DII was negatively correlated with both global cognitive scores and specific domains like memory and executive function (132). While some studies suggest gender differences—for instance, a more pronounced E-DII association with processing speed in men mediated by gamma-glutamyl transferase (133)—other NHANES analyses found no significant gender interaction (127).

Prospective data from the UK Biobank, involving over 160,000 participants, show that high DII diets are significantly associated with increased risks of all-cause dementia and AD (134). When combined with genetic susceptibility, high DII scores significantly escalate dementia risk, particularly among APOE ε4 carriers (135). Furthermore, the Framingham Heart Study offspring cohort found that participants with high DII scores had a 21% increased risk of all-cause dementia and a 20% increased risk of AD over 13.1 years (136). The Greek HELIAD study confirmed that each unit increase in the DII increases dementia risk by 21%, with the most pro-inflammatory group facing triple the risk of the lowest group (137). Beyond incidence, high DII scores are associated with increased AD-related mortality, while an anti-inflammatory diet is linked to lower AD-specific mortality, highlighting the potential of dietary intervention to improve prognosis (138, 139).

#### 5.7.2 Critical appraisal and the risk of reverse causation

A 2025 comprehensive meta-analysis of prospective cohort studies reinforces that a high dietary inflammatory potential significantly increases the risk of cognitive impairment (140). Despite this strong statistical association, translating these findings into a unidirectional causal framework requires a critical lens regarding reverse causation. In the prodromal stages of neurodegeneration (T3DM), patients frequently experience olfactory decline, impaired executive function, and mood alterations. These neurological changes can compel individuals to shift their dietary patterns away from complex anti-inflammatory foods toward highly processed, pro-inflammatory “comfort foods.” Thus, a high DII score in shorter follow-up studies might partially represent a behavioral consequence, rather than the sole etiology, of early brain aging.

#### 5.7.3 Imaging evidence of structural brain changes and biological aging

Advances in neuroimaging have allowed clinical research to explore the objective links between the DII, structural brain changes, and biological brain age. MRI analysis from the Framingham Heart Study linked high DII scores to smaller total brain volume and total gray matter volume, along with larger lateral ventricular volume, suggesting that pro-inflammatory diets accelerate brain atrophy (141). UK Biobank research further found that among older adults with cardiometabolic diseases (including diabetes), those adhering to low DII diets had significantly larger gray matter volumes and smaller white matter hyperintensity (WMH) volumes, suggesting that anti-inflammatory diets maintain cerebrovascular integrity and reduce neurodegeneration (142). Furthermore, machine learning-based “brain age” studies show that high DII scores are significantly associated with an increased Brain Age Gap (BAG), indicating that dietary inflammation accelerates the biological aging process of the brain (143).

#### 5.7.4 EDIP and cognitive health

The EDIP have also been applied in cognitive research. A longitudinal analysis of the Framingham Heart Study offspring cohort found that cumulative DII scores were associated with an accelerated decline in processing speed and executive function (Trail Making Test B-A) (144). In the Nurses’ Health Study, however, EDIP scores were not directly associated with poorer cognitive performance, suggesting that different dietary indices may capture cognition-related dietary features differently (145). Cross-sectional research on patients with vascular dementia found that while these patients tended to consume more pro-inflammatory foods (e.g., fried foods), the association between the EDIP and vascular dementia was not statistically significant after adjusting for confounders (146).

#### 5.7.5 Metabolic and inflammatory mediation mechanisms under the T3DM perspective

Clinical research has moved beyond direct associations to explore the mediating roles of metabolic disturbances and inflammation—the core features of T3DM—in the link between the DII and cognitive outcomes. Glucose metabolism indicators have been identified as key mediators. In non-diabetic older adults, glycated hemoglobin (HbA1c) partially mediates the association between the E-DII and cognitive test scores. In diabetic patients, however, the direct toxicity of hyperglycemia may mask this mediation, suggesting that dietary inflammation impairs cognitive function by disrupting long-term glycemic homeostasis (129). Furthermore, in individuals with existing cardiometabolic disease, low DII diets exert a particularly pronounced effect on reducing dementia risk, emphasizing the importance of controlling dietary inflammation in high-risk metabolic states (142). Systemic inflammatory markers represent a direct pathway; high DII scores correlate with elevated serum CRP and IL-6 levels, which in turn predict cognitive decline (147). In the UK Biobank, a systemic inflammation score (INFLA-score) mediated approximately 8% of the DII-BAG association (143). Finally, markers of cellular senescence, such as leukocyte telomere length (LTL) and mitochondrial DNA copy number (mtDNAcn), have been found to mediate the association between the DII and mild cognitive impairment risk, suggesting that pro-inflammatory diets lead to neuronal dysfunction by accelerating cellular aging and energy metabolism disorders (148).

### 5.8 Clinical research status of the FII in metabolic and related health

The FISI score, developed from the FII framework as a novel food-based inflammatory assessment tool, has demonstrated robust pathological correlations in multiple clinical studies. Research confirms that FISI scores are significantly positively correlated with systemic inflammatory biomarkers such as high-sensitivity C-reactive protein (hs-CRP) (29). During the development of the FII, Wang et al. utilized the USDA-FCT database for deep validation and concurrently introduced the China-FCT for comparative cross-validation. The results confirmed that the FII’s assessment trends for the inflammatory potential of typical food groups (e.g., oils, whole grains) are highly consistent across Eastern and Western dietary contexts. In population cohorts, high FISI scores are positively associated with the prevalence of MASLD. Furthermore, Tao et al. systematically demonstrated that FISI34, FISI26-USDA, and FISI26-CHINA are closely linked to the increased prevalence of sarcopenia (149).

Recently, emerging clinical evidence has begun to highlight the potential advantages of the FII in metabolic contexts. A 2025 cross-sectional study based on the large-scale NHANES cohort (150) demonstrated a linear dose–response relationship between higher FII scores and increased risk of metabolic dysfunction-associated steatotic liver disease (MASLD). Notably, compared to the traditional DII, the FII significantly improved risk reclassification (Net Reclassification Improvement [NRI] = 0.0556, *p* = 0.002) and discrimination (150).

However, we must objectively acknowledge that direct longitudinal cohort studies utilizing the FII and FISI in the specific continuum of MetS, T2DM, and AD remain sparse. Consequently, while the FII effectively overcomes the clinical translation bottleneck conceptually, it currently serves as a highly promising, early-stage index rather than an extensively validated prognostic tool. Future prospective research is urgently needed to fully explore its early warning value in high-risk T3DM populations.

## 6 Discussion and future perspectives

### 6.1 Clinical translation and precision interventions of the FII framework

The transition from the DII to the FII/FISI framework marks a critical leap toward precision nutrition. However, a balanced perspective is essential when evaluating these methodologies. While the FII offers superior granularity by accounting for the ‘food matrix effect’ and providing actionable clinical advice, the DII remains irreplaceable for large-scale historical research. Due to its foundation in globally standardized nutrient databases and extensive validation across hundreds of international cohorts, the DII continues to be the gold standard for long-term retrospective studies and cross-cultural epidemiological comparisons. Therefore, the FII should be viewed not as a total replacement for the DII, but as a high-resolution clinical evolution designed to bridge the gap between population-level research and individualized therapeutic intervention. The emergence of FII and FISI, represents more than a methodological simplification; it signifies a fundamental reconstruction of clinical application scenarios. By quantifying intra-group heterogeneity through the FII, clinical practitioners and registered dietitians are no longer restricted to generic advice, such as “reducing total fat intake.” Instead, they can precisely identify specific anti-inflammatory substitution strategies based on the food matrix effect. As illustrated in Table 2, the FII enables a transition from abstract nutrient-centric views to actionable clinical substitutions—such as replacing pro-inflammatory palm oil with anti-inflammatory flaxseed oil or substituting refined wheat with antioxidant-rich black rice—thereby directly modulating the pathophysiological pathways of chronic diseases like T3DM. As envisioned by Hébert et al. (32), the ultimate objective of dietary assessment indices is to facilitate robust clinical decision support. By integrating circulating biomarkers, such as hs-CRP and HbA1c, clinicians can establish individualized dietary inflammatory thresholds, marking a transition from general population guidelines to personalized nutritional prescriptions (147, 151, 152). As shown in the lower portion of Figure 1, embedding FII algorithms into digital health terminals allows for real-time tracking of patient FISI, enabling timely correction of isolated adverse dietary behaviors that might otherwise trigger systemic inflammatory cascades (153).

**Table 2:** FII-based food substitution model and pathophysiological impact on MetS and T3DM.

Food group	Nutrient-centric view (traditional DII)	Food matrix advantage (FII/FISI)	Clinical substitution	Pathophysiological impact on M	Refs
Vegetable Oils	Focus on total unsaturated fats.	Distinguishes flaxseed (anti-inf.) from Palm (pro-inf.) oils.	Refined blended oils → flaxseed oil	Reduces circulating FFAs; lowers BBB oxidative stress.	(161)
Seafood	Regarded as anti-inflammatory due to n-3.	Quantifies DHA/EPA density gradients between species.	Lean white fish → Mackerel/Salmon	High n-3 concentration promotes microglial M2 polarization.	(162)
Vegetables	Binary scoring (all vegetables = positive).	Decodes matrix: water content vs. flavonoid potency.	High-water veg → dark cruciferous veg	Flavonoids inhibit JNK pathway; restore insulin signaling.	(163)
Grains	Statistical focus on fiber content.	Evaluates antioxidant depletion during refining.	Refined wheat → black rice/oats	Stabilizes glucose; prevents insulin-driven signal blockade.	(164, 165)
Spices	Often ignored as minor components.	Identifies high FISI weight at trace dosages.	Regular intake → turmeric/ginger	Curcumin crosses to reduce Aβ plaque deposition.	(166, 167)

DII, Dietary Inflammatory Index; FII, Food Inflammation Index; FISI, Food Inflammation Scores of Individuals; BBB, Blood–Brain Barrier; JNK, c-Jun N-terminal kinase.

As Machine Learning (ML) algorithms reach maturity, researchers have begun utilizing big-data-driven models to optimize weight allocation across food groups, facilitating risk prediction for complex conditions such as CVD (153). Furthermore, the performance of Large Language Models (LLMs), including ChatGPT and DeepSeek (154, 155), in content generation offers a novel pathway for the clinical implementation of the FII. Although current artificial intelligence (AI) systems still require human oversight regarding the precision of professional nutritional calculations, their potential for generating feasible anti-inflammatory dietary plans is already evident. By integrating FII algorithms into digital health applications, dietitians can monitor dietary inflammatory dynamics in real-time, facilitating truly dynamic and precision interventions.

In the future, the further refinement of the FII framework will depend on the enrichment of global Food Composition Tables (FCTs) and validation across diverse populations. Critically, current research must address the blind spot of ‘culinary matrix variability.’ The inflammatory potential of food is not a static attribute but is dynamically reshaped by processing; for instance, high-heat frying can trigger the formation of pro-inflammatory AGEs (156), whereas probiotic fermentation may enrich anti-inflammatory metabolites (157). Furthermore, to overcome existing data silos, the integration of FISI algorithms into Digital Therapeutics (DTx) platforms is essential (153). This transition will facilitate a real-time, closed-loop feedback system, shifting dietary management from retrospective assessment to proactive, precision intervention. Additionally, we propose that derivative tools conceptualized within the FII theoretical framework—such as energy-adjusted FISI (E-FISI), Children’s FISI (C-FISI), and Pregnancy FISI (P-FISI)—represent promising directions for future research. Although these indices have yet to be empirically validated, their development would significantly enhance precision in clinical interventions.

### 6.2 Critical limitations and future challenges of dietary inflammatory indices

While the transition from DII to FII represents a pivotal methodological evolution, several inherent limitations in assessing dietary inflammation must be critically addressed.

First, the blind spot of microbiome heterogeneity: Both traditional nutrient-based (DII) and novel food-based (FII) indices assign static inflammatory scores to diets. However, top-tier evidence demonstrates that the physiological impact of a diet is fundamentally mediated by an individual’s unique gut microbiota architecture (48). The same “anti-inflammatory” food matrix (e.g., high-fiber vegetables) may yield vastly different systemic inflammatory responses depending on the host’s microbial capacity to ferment it into short-chain fatty acids (SCFAs). Current indices fail to capture this highly individualized host-microbiome interaction.

Second, inherent assessment and database biases: The FII is intrinsically dependent on regional Food Composition Tables (FCTs) and relies on subjective dietary recalls. A recent scoping review highlighted that these databases often lag behind the rapid evolution of modern ultra-processed foods and fail to account for variations in inflammatory potential caused by highly variable culinary processing methods (e.g., high-heat frying versus steaming) or agricultural differences (83).

Lastly, polypharmacy in aging populations: In older adults suffering from MetS or early-stage T3DM, polypharmacy is highly prevalent. The subtle inflammatory modulation achieved by precision nutrition might be heavily masked or complexly modified by the widespread use of potent pharmacological agents (e.g., statins, metformin). Disentangling the pure dietary effect from medication interactions remains a substantial hurdle for the clinical implementation of FISI.

## 7 Conclusion

In conclusion, the evolution of dietary inflammatory assessment from nutrient-centric indices to food-based logic marks a pivotal advancement in precision nutrition. By bridging the mechanistic gap along the gut-metabolism-brain axis, this review underscores chronic low-grade inflammation as the core driver in the pathological progression from MetS to T3DM. The FII and FISI frameworks, by effectively capturing the whole-food matrix effect, offer a promising approach to address the clinical translation bottlenecks inherent in previous methodologies. When integrated with digital health technologies and tailored to critical life stages—such as through the proposed C-FISI and P-FISI indices—these tools offer a robust foundation for personalized, pre-emptive interventions. Ultimately, this methodological shift will empower clinicians and researchers to develop more effective, evidence-based strategies to mitigate the global burden of neuro-metabolic chronic diseases.

## References

[1] Neeland IJ Lim S Tchernof A Gastaldelli A Rangaswami J Ndumele CE . Metabolic syndrome. Nat Rev Dis Primers. (2024) 10:77. doi: 10.1038/s41572-024-00563-5 39420195

[2] Saklayen MG. The global epidemic of the metabolic syndrome. Curr Hypertens Rep. (2018) 20:12. doi: 10.1007/s11906-018-0812-z, 29480368 PMC5866840

[3] Tune JD Goodwill AG Sassoon DJ Mather KJ. Cardiovascular consequences of metabolic syndrome. Transla Res. (2017) 183:57–70. doi: 10.1016/j.trsl.2017.01.001, 28130064 PMC5393930

[4] Alberti KG Zimmet P Shaw J. Metabolic syndrome—a new world‐wide definition. A consensus statement from the international diabetes federation. Diabet Med. (2006) 23:469–80. doi: 10.1111/j.1464-5491.2006.01858.x, 16681555

[5] Grinshpan LS Eilat-Adar S Ivancovsky-Wajcman D Kariv R Gillon-Keren M Zelber-Sagi S. Ultra-processed food consumption and non-alcoholic fatty liver disease, metabolic syndrome and insulin resistance: a systematic review. JHEP Rep. (2024) 6:100964. doi: 10.1016/j.jhepr.2023.100964, 38234408 PMC10792654

[6] Kaykhaei MA Shahraki E Motamedi M Ansari-Moghaddam A Mohammadi M Fatideh TM. Metabolic syndrome in patients with chronic kidney disease. Clinic Transl Metab. (2024) 22:9. doi: 10.1007/s12018-024-09298-z

[7] Helvaci N Yildiz BO. Polycystic ovary syndrome as a metabolic disease. Nat Rev Endocrinol. (2025) 21:230–44. doi: 10.1038/s41574-024-01057-w 39609634

[8] Furukawa S Fujita T Shimabukuro M Iwaki M Yamada Y Nakajima Y . Increased oxidative stress in obesity and its impact on metabolic syndrome. J Clin Invest. (2004) 114:1752–61. doi: 10.1172/JCI21625, 15599400 PMC535065

[9] Wang W Chen ZY Guo XL Tu M. Monocyte to high-density lipoprotein and apolipoprotein A1 ratios: novel indicators for metabolic syndrome in Chinese newly diagnosed type 2 diabetes. Front Endocrinol. (2022) 13:935776. doi: 10.3389/fendo.2022.935776 PMC9330493 35909551

[10] Hotamisligil GS. Inflammation and metabolic disorders. Nature. (2006) 444:860–7. doi: 10.1038/nature05485 17167474

[11] Ajoolabady A Pratico D Lin L Mantzoros CS Bahijri S Tuomilehto J . Inflammation in atherosclerosis: pathophysiology and mechanisms. Cell Death Dis. (2024) 15:817. doi: 10.1038/s41419-024-07166-8, 39528464 PMC11555284

[12] Jiang H Zhou Y Nabavi SM Sahebkar A Little PJ Xu S . Mechanisms of oxidized LDL-mediated endothelial dysfunction and its consequences for the development of atherosclerosis. Front Cardiovasc Med. (2022) 9:925923. doi: 10.3389/fcvm.2022.925923, 35722128 PMC9199460

[13] Botella Lucena P Heneka MT. Inflammatory aspects of Alzheimer's disease. Acta Neuropathol. (2024) 148:31. doi: 10.1007/s00401-024-02790-2 39196440

[14] Selkoe DJ Hardy J. The amyloid hypothesis of Alzheimer's disease at 25 years. EMBO Mol Med. (2016) 8:595–608. doi: 10.15252/emmm.201606210, 27025652 PMC4888851

[15] Sędzikowska A Szablewski L. Insulin and insulin resistance in Alzheimer's disease. Int J Mol Sci. (2021) 22:987. doi: 10.3390/ijms22189987, 34576151 PMC8472298

[16] Dewanjee S Chakraborty P Bhattacharya H Chacko L Singh B Chaudhary A . Altered glucose metabolism in Alzheimer's disease: role of mitochondrial dysfunction and oxidative stress. Free Radic Biol Med. (2022) 193:134–57. doi: 10.1016/j.freeradbiomed.2022.09.032, 36206930

[17] de la Monte SM Wands JR. Alzheimer's disease is type 3 diabetes-evidence reviewed. J Diabetes Sci Technol. (2008) 2:1101–13. doi: 10.1177/193229680800200619, 19885299 PMC2769828

[18] Kellar D Craft S. Brain insulin resistance in Alzheimer's disease and related disorders: mechanisms and therapeutic approaches. Lancet Neurol. (2020) 19:758–66. doi: 10.1016/S1474-4422(20)30231-3, 32730766 PMC9661919

[19] Kacem H d'Angelo M Qosja E Topi S Castelli V Cimini A. The inflammatory bridge between type 2 diabetes and neurodegeneration: a molecular perspective. Int J Mol Sci. (2025) 26:566. doi: 10.3390/ijms26157566, 40806709 PMC12347821

[20] Chen Y He Y Han J Wei W Chen F. Blood-brain barrier dysfunction and Alzheimer's disease: associations, pathogenic mechanisms, and therapeutic potential. Front Aging Neurosci. (2023) 15:1258640. doi: 10.3389/fnagi.2023.1258640, 38020775 PMC10679748

[21] Gopinath A Mackie PM Phan LT Tansey MG Khoshbouei H. The complex role of inflammation and gliotransmitters in Parkinson's disease. Neurobiol Dis. (2023) 176:105940. doi: 10.1016/j.nbd.2022.105940, 36470499 PMC10372760

[22] Michailidis M Moraitou D Tata DA Kalinderi K Papamitsou T Papaliagkas V. Alzheimer's disease as type 3 diabetes: common pathophysiological mechanisms between Alzheimer's disease and type 2 diabetes. Int J Mol Sci. (2022) 23:687. doi: 10.3390/ijms23052687, 35269827 PMC8910482

[23] Berisha H Hattab R Comi L Giglione C Migliaccio S Magni P. Nutrition and lifestyle interventions in managing Dyslipidemia and cardiometabolic risk. Nutrients. (2025) 17:776. doi: 10.3390/nu17050776, 40077646 PMC11902110

[24] Shivappa N Steck SE Hurley TG Hussey JR Hébert JR. Designing and developing a literature-derived, population-based dietary inflammatory index. Public Health Nutr. (2014) 17:1689–96. doi: 10.1017/S1368980013002115, 23941862 PMC3925198

[25] Canto-Osorio F Denova-Gutierrez E Sánchez-Romero LM Salmerón J Barrientos-Gutierrez T. Dietary inflammatory index and metabolic syndrome in Mexican adult population. Am J Clin Nutr. (2020) 112:373–80. doi: 10.1093/ajcn/nqaa135, 32511694

[26] Ariya M Shahraki HR Farjam M Ehrampoush E Bahramali E Homayounfar R . Dietary inflammatory index and metabolic syndrome in Iranian population (Fasa Persian cohort study). Sci Rep. (2020) 10:16762. doi: 10.1038/s41598-020-73844-0, 33028906 PMC7542151

[27] Li R Zhan W Huang X Zhang Z Zhou M Bao W . Association of dietary inflammatory index and metabolic syndrome in the elderly over 55 years in northern China. Br J Nutr. (2021) 128:1082–9. doi: 10.1017/S0007114521004207 34658314 PMC9381302

[28] Laouali N Mancini FR Hajji-Louati M El Fatouhi D Balkau B Boutron-Ruault MC . Dietary inflammatory index and type 2 diabetes risk in a prospective cohort of 70,991 women followed for 20 years: the mediating role of BMI. Diabetologia. (2019) 62:2222–32. doi: 10.1007/s00125-019-04972-0, 31396661

[29] Wang Z Yuan C Zhang Y Abdelaty NS Chen C Shen J . Food inflammation index reveals the key inflammatory components in foods and heterogeneity within food groups: how do we choose food? J Adv Res. (2025) 74:87–98. doi: 10.1016/j.jare.2024.10.010, 39401693 PMC12302825

[30] Bang HO Dyerberg J Sinclair HM. The composition of the Eskimo food in north western Greenland. Am J Clin Nutr. (1980) 33:2657–61. doi: 10.1093/ajcn/33.12.2657, 7435433

[31] Pauling L. Evolution and the need for ascorbic acid. Proc Natl Acad Sci USA. (1970) 67:1643–8. doi: 10.1073/pnas.67.4.1643, 5275366 PMC283405

[32] Hébert JR Shivappa N Wirth MD Hussey JR Hurley TG. Perspective: the dietary inflammatory index (DII)-lessons learned, improvements made, and future directions. Adv Nutr. (2019) 10:185–95. doi: 10.1093/advances/nmy071, 30615051 PMC6416047

[33] Chen Y Luo Z Cheng L Wang Q Zou F Warsi MA . Development and validation of the China dietary inflammatory index (CHINA-DII). Nutrients. (2025) 17:687. doi: 10.3390/nu17101687 40431426 PMC12114556

[34] Tabung FK Smith-Warner SA Chavarro JE Wu K Fuchs CS Hu FB . Development and validation of an empirical dietary inflammatory index. J Nutr. (2016) 146:1560–70. doi: 10.3945/jn.115.228718, 27358416 PMC4958288

[35] Byrd DA Judd SE Flanders WD Hartman TJ Fedirko V Bostick RM. Development and validation of novel dietary and lifestyle inflammation scores. J Nutr. (2019) 149:2206–18. doi: 10.1093/jn/nxz165, 31373368 PMC6887697

[36] Szabo Z Koczka V Marosvolgyi T Szabo E Frank E Polyak E . Possible biochemical processes underlying the positive health effects of plant-based diets-a narrative review. Nutrients. (2021) 13:593. doi: 10.3390/nu13082593, 34444753 PMC8398942

[37] Lee DH Li J Li Y Liu G Wu K Bhupathiraju S . Dietary inflammatory and insulinemic potential and risk of type 2 diabetes: results from three prospective U.S. cohort studies. Diabetes Care. (2020) 43:2675–83. doi: 10.2337/dc20-0815, 32873589 PMC7576428

[38] Navalón-Monllor V Soriano-Romaní L Silva M de las Hazas N Hernando-Quintana TSD Esteve PM . Microbiota dysbiosis caused by dietetic patterns as a promoter of Alzheimer's disease through metabolic syndrome mechanisms. Food Funct. (2023) 14:7317–34. doi: 10.1039/d3fo01257c 37470232

[39] Zhao Q Tan X Su Z Manzi HP Su L Tang Z . The relationship between the dietary inflammatory index (DII) and metabolic syndrome (MetS) in middle-aged and elderly individuals in the United States. Nutrients. (2023) 15:857. doi: 10.3390/nu15081857, 37111075 PMC10146265

[40] Thomas MS Blesso CN Calle MC Chun OK Puglisi M Fernandez ML. Dietary influences on gut microbiota with a focus on metabolic syndrome. Metab Syndr Relat Disord. (2022) 20:429–39. doi: 10.1089/met.2021.0131, 35704900

[41] Tian Y Fu M Su J Yan M Yu J Wang C . Gut microbiota dysbiosis and intestinal barrier impairment in diarrhea caused by cold drink and high-fat diet. Toxicology. (2024) 502:153728. doi: 10.1016/j.tox.2024.153728, 38216112

[42] Sonier B Patrick C Ajjikuttira P Scott FW. Intestinal immune regulation as a potential diet-modifiable feature of gut inflammation and autoimmunity. Int Rev Immunol. (2009) 28:414–45. doi: 10.3109/08830180903208329, 19954357

[43] Nagpal R Yadav H. Bacterial translocation from the gut to the distant organs: an overview. Ann Nutr Metab. (2017) 71:11–6. doi: 10.1159/000479918 28950279

[44] Mohammad S Thiemermann C. Role of metabolic endotoxemia in systemic inflammation and potential interventions. Front Immunol. (2020) 11:594150. doi: 10.3389/fimmu.2020.594150 33505393 PMC7829348

[45] Velloso LA Folli F Saad MJ. TLR4 at the crossroads of nutrients, gut microbiota, and metabolic inflammation. Endocr Rev. (2015) 36:245–71. doi: 10.1210/er.2014-1100, 25811237

[46] Valle J. Biofilm-associated proteins: from the gut biofilms to neurodegeneration. Gut Microbes. (2025) 17:2461721. doi: 10.1080/19490976.2025.2461721, 39898557 PMC11792866

[47] Kowalski K Mulak A. Brain-gut-microbiota Axis in Alzheimer's disease. J Neurogastroenterol Motil. (2019) 25:48–60. doi: 10.5056/jnm18087, 30646475 PMC6326209

[48] Lozano CP Wilkens LR Shvetsov YB Maskarinec G Park SY Shepherd JA . Associations of the dietary inflammatory index with total adiposity and ectopic fat through the gut microbiota, LPS, and C-reactive protein in the multiethnic cohort-adiposity phenotype study. Am J Clin Nutr. (2022) 115:1344–56. doi: 10.1093/ajcn/nqab398, 34871345 PMC9071464

[49] Liu J Zhang Y Li X Hou Z Wang B Chen L . Stratified dietary inflammatory potential identifies oral and gut microbiota differences associated with cognitive function in older adults. Sci Rep. (2025) 15:18988. doi: 10.1038/s41598-025-02292-5, 40447628 PMC12125347

[50] McArdle MA Finucane OM Connaughton RM McMorrow AM Roche HM. Mechanisms of obesity-induced inflammation and insulin resistance: insights into the emerging role of nutritional strategies. Front Endocrinol. (2013) 4:52. doi: 10.3389/fendo.2013.00052 PMC3650620 23675368

[51] Suganami T Tanaka M Ogawa Y. Adipose tissue inflammation and ectopic lipid accumulation. Endocr J. (2012) 59:849–57. doi: 10.1507/endocrj.EJ12-0271 22878669

[52] Ghazarian M Luck H Revelo XS Winer S Winer DA. Immunopathology of adipose tissue during metabolic syndrome. Turk Patoloji Derg. (2015) 31:172–80. doi: 10.5146/tjpath.2015.01323 26177326

[53] Wang S Shi Y Xin R Kang H Xiong H Ren J. Exploring the role of insulin resistance in bridging the metabolic syndrome and Alzheimer's disease-a review of mechanistic studies. Front Endocrinol (Lausanne). (2025) 16:1614006. doi: 10.3389/fendo.2025.1614006, 41133228 PMC12540164

[54] Rondinone CM Wang LM Lonnroth P Wesslau C Pierce JH Smith U. Insulin receptor substrate (IRS) 1 is reduced and IRS-2 is the main docking protein for phosphatidylinositol 3-kinase in adipocytes from subjects with non-insulin-dependent diabetes mellitus. Proc Natl Acad Sci USA. (1997) 94:4171–5. doi: 10.1073/pnas.94.8.4171, 9108124 PMC20591

[55] Solinas G Becattini B. JNK at the crossroad of obesity, insulin resistance, and cell stress response. Mol Metab. (2017) 6:174–84. doi: 10.1016/j.molmet.2016.12.001, 28180059 PMC5279903

[56] Feng L Huang F Ma Y Tang J. The effect of high-fat diet and exercise intervention on the TNF-α level in rat spleen. Front Immunol. (2021) 12:671167. doi: 10.3389/fimmu.2021.671167, 34975827 PMC8714663

[57] Li M Chi X Wang Y Setrerrahmane S Xie W Xu H. Trends in insulin resistance: insights into mechanisms and therapeutic strategy. Signal Transduct Target Ther. (2022) 7:216. doi: 10.1038/s41392-022-01073-0, 35794109 PMC9259665

[58] Savova MS Mihaylova LV Tews D Wabitsch M Georgiev MI. Targeting PI3K/AKT signaling pathway in obesity. Biomed Pharmacother. (2023) 159:114244. doi: 10.1016/j.biopha.2023.114244 36638594

[59] Sylow L Kleinert M Pehmøller C Prats C Chiu TT Klip A . Akt and Rac1 signaling are jointly required for insulin-stimulated glucose uptake in skeletal muscle and downregulated in insulin resistance. Cell Signal. (2014) 26:323–31. doi: 10.1016/j.cellsig.2013.11.007, 24216610

[60] Erichsen JM Fadel JR Reagan LP. Peripheral versus central insulin and leptin resistance: role in metabolic disorders, cognition, and neuropsychiatric diseases. Neuropharmacology. (2022) 203:108877. doi: 10.1016/j.neuropharm.2021.108877, 34762922 PMC8642294

[61] Xue B Zhou A Zhong Y Mao Y Peng R Chen Y . MMP-9 regulates disulphide isomerase activity of TGM2 to enhance fusion glycoprotein-mediated syncytium formation of respiratory syncytial virus. Protein. Cell. (2025) 17:59–76. doi: 10.1093/procel/pwaf063 PMC12888920 40808571

[62] Wilhelmus MM Otte-Höller I van Triel JJ Veerhuis R Maat-Schieman ML Bu G . Lipoprotein receptor-related protein-1 mediates amyloid-beta-mediated cell death of cerebrovascular cells. Am J Pathol. (2007) 171:1989–99. doi: 10.2353/ajpath.2007.070050, 18055545 PMC2111121

[63] Zhao Y Li D Zhao J Song J Zhao Y. The role of the low-density lipoprotein receptor-related protein 1 (LRP-1) in regulating blood-brain barrier integrity. Rev Neurosci. (2016) 27:623–34. doi: 10.1515/revneuro-2015-0069, 27206317

[64] Steen E Terry BM Rivera EJ Cannon JL Neely TR Tavares R . Impaired insulin and insulin-like growth factor expression and signaling mechanisms in Alzheimer's disease – is this type 3 diabetes? J Alzheimer's Dis. (2005) 7:63–80. doi: 10.3233/JAD-2005-7107, 15750215

[65] Beharry C Cohen LS Di J Ibrahim K Briffa-Mirabella S Alonso Adel C. Tau-induced neurodegeneration: mechanisms and targets. Neurosci Bull. (2014) 30:346–58. doi: 10.1007/s12264-013-1414-z, 24733656 PMC5562659

[66] Pu T Zou W Feng W Zhang Y Wang L Wang H . Persistent malfunction of glymphatic and meningeal lymphatic drainage in a mouse model of subarachnoid Hemorrhage. Exp Neurobiol. (2019) 28:104–18. doi: 10.5607/en.2019.28.1.104, 30853828 PMC6401547

[67] Noubiap JJ Nansseu JR Nyaga UF Ndoadoumgue AL Ngouo AT Tounouga DN . Worldwide trends in metabolic syndrome from 2000 to 2023: a systematic review and modelling analysis. Nat Commun. (2025) 17:573. doi: 10.1038/s41467-025-67268-5, 41350289 PMC12808104

[68] Mazidi M Shivappa N Wirth MD Hebert JR Mikhailidis DP Kengne AP . Dietary inflammatory index and cardiometabolic risk in US adults. Atherosclerosis. (2018) 276:23–7. doi: 10.1016/j.atherosclerosis.2018.02.020 30015256

[69] Marosvolgyi T Szabo E Erhardt E Molnár D Decsi T. High contribution of palmitoleic acid to plasma lipid classes in obese children with and without metabolic risk factors related to obesity. J Pediatr Gastroenterol Nutr. (2006) 42:764. doi: 10.1002/j.1536-4801.2006.tb01764.x

[70] Ren Z Zhao A Wang Y Meng L Szeto IM Li T . Association between dietary inflammatory index, C-reactive protein and metabolic syndrome: a cross-sectional study. Nutrients. (2018) 10:831. doi: 10.3390/nu10070831, 29954070 PMC6073906

[71] Kenđel Jovanović G Pavičić Žeželj S Klobučar Majanović S Mrakovcic-Sutic I Šutić I. Metabolic syndrome and its association with the dietary inflammatory index (DII)®in a Croatian working population. J Hum Nutr Diet. (2020) 33:128–37. doi: 10.1111/jhn.12695, 31602707

[72] Neufcourt L Assmann KE Fezeu LK Touvier M Graffouillère L Shivappa N . Prospective association between the dietary inflammatory index and metabolic syndrome: findings from the SU.VI.MAX study. Nutr Metab Cardiovasc Dis. (2015) 25:988–96. doi: 10.1016/j.numecd.2015.09.002, 26482566

[73] Alkerwi A Shivappa N Crichton G Hébert JR. No significant independent relationships with cardiometabolic biomarkers were detected in the observation of cardiovascular risk factors in Luxembourg study population. Nutr Res. (2014) 34:1058–65. doi: 10.1016/j.nutres.2014.07.017, 25190219 PMC4329249

[74] Pourmontaseri H Sepehrinia M Kuchay MS Farjam M Vahid F Dehghan A . The association between energy-adjusted dietary inflammatory index and metabolic syndrome and its mediatory role for cardiometabolic diseases: a prospective cohort study. Front Nutr. (2024) 11:1429883. doi: 10.3389/fnut.2024.1429883, 39161908 PMC11330808

[75] Abdollahzad H Pasdar Y Nachvak SM Rezaeian S Saber A Nazari R. The relationship between the dietary inflammatory index and metabolic syndrome in Ravansar cohort study. Diabetes Metab Syndr Obes. (2020) 13:477–87. doi: 10.2147/DMSO.S240641, 32110080 PMC7041598

[76] Anjom-Shoae J Namazi N Ayati MH Darbandi M Najafi F Pasdar Y. Dietary insulin index and load in relation to cardiometabolic risk factors in patients with type 2 diabetes mellitus: a cross-sectional study on the RaNCD cohort study. Nutrition. (2023) 105:111830. doi: 10.1016/j.nut.2022.111830, 36252460

[77] Shakeri Z Mirmiran P Khalili-Moghadam S Hosseini-Esfahani F Ataie-Jafari A Azizi F. Empirical dietary inflammatory pattern and risk of metabolic syndrome and its components: Tehran lipid and glucose study. Diabetol Metab Syndr. (2019) 11:16. doi: 10.1186/s13098-019-0411-4, 30805034 PMC6373046

[78] Khan I Kwon M Shivappa N Kim MK. Proinflammatory dietary intake is associated with increased risk of metabolic syndrome and its components: results from the population-based prospective study. Nutrients. (2020) 12:196. doi: 10.3390/nu12041196, 32344617 PMC7230546

[79] Stemerdink NC Wesselink E Lai JS Loh J van Dam RM Sim X . An empirical dietary inflammatory pattern increases the incidence of the metabolic syndrome in a multi-ethnic Asian population. Eur J Nutr. (2025) 64:256. doi: 10.1007/s00394-025-03770-2, 40810800 PMC12354496

[80] Gallardo-Alfaro L Bibiloni MDM Bouzas C Mascaró CM Martínez-González M Salas-Salvadó J . Physical activity and metabolic syndrome severity among older adults at cardiovascular risk: 1-year trends. Nutr Metab Cardiovasc Dis. (2021) 31:2870–86. doi: 10.1016/j.numecd.2021.06.015, 34366176

[81] Marx W Veronese N Kelly JT Smith L Hockey M Collins S . The dietary inflammatory index and human health: an umbrella review of Meta-analyses of observational studies. Adv Nutr. (2021) 12:1681–90. doi: 10.1093/advances/nmab037, 33873204 PMC8483957

[82] Linton C Schaumberg MA Wright HH. Convergent validity of multiple 24-h dietary recalls and food frequency questionnaire in calculating the dietary inflammatory index score in community-dwelling older adults. Food Sci Nutr. (2026) 14:e71501. doi: 10.1002/fsn3.71501, 41625270 PMC12856060

[83] El-Khoury C Jannasch F Schulze MB. Scoping review of dietary quality indices: heterogeneity of definitions and health associations among adults. Nutr Rev. (2025). doi: 10.1093/nutrit/nuaf231, 41317033 PMC13659007

[84] Noorzae R Bjerregaard AA Halldorsson TI Granström C Brantsæter AL Borge T . Association between a pro-inflammatory dietary pattern during pregnancy and type 1 diabetes risk in offspring: prospective cohort study. J Epidemiol Community Health. (2025) 79:737–45. doi: 10.1136/jech-2024-223320, 40592515

[85] Panagos PG Vishwanathan R Penfield-Cyr A Matthan NR Shivappa N Wirth MD . Breastmilk from obese mothers has pro-inflammatory properties and decreased neuroprotective factors. J Perinatol. (2016) 36:284–90. doi: 10.1038/jp.2015.199, 26741571 PMC4888773

[86] Betanzos-Robledo L Rodríguez-Carmona Y Contreras-Manzano A Lamadrid-Figueroa H Jansen E Tellez-Rojo MM . Greater cumulative exposure to a pro-inflammatory diet is associated with higher metabolic syndrome score and blood pressure in young Mexican adults. Nutr Res. (2020) 81:81–9. doi: 10.1016/j.nutres.2020.08.005, 32942060 PMC9900665

[87] Wang Y Armijos RX Weigel MM. Dietary inflammatory index and cardiometabolic risk in Ecuadorian school-age children. J Am Nutr Assoc. (2023) 42:618–27. doi: 10.1080/27697061.2022.2113177, 35980812

[88] Leite LO Lira CRN Pitangueira JCD Costa PRF. Dietary inflammatory index, body adiposity indicators and blood pressure in children and adolescents: a systematic review and meta-analysis. Nutr Res Rev. (2025) 38:944–60. doi: 10.1017/S0954422425100164, 40856259

[89] Behrooz M Hajjarzadeh S Moludi J Bakhshimoghaddam F Ostadrahimi A. Association between the dietary inflammatory index and spexin levels, metabolic syndrome, and inflammatory biomarkers in children with obesity. J Diabetes Metab Disord. (2025) 24:262. doi: 10.1007/s40200-025-01782-7, 41210113 PMC12594623

[90] Seremet Kurklu N Karatas Torun N Ozen Kucukcetin I Akyol A. Is there a relationship between the dietary inflammatory index and metabolic syndrome among adolescents? J Pediatr Endocrinol Metab. (2020) 33:495–502. doi: 10.1515/jpem-2019-0409, 32084004

[91] Çağiran Yilmaz F Açık M. Children-dietary inflammatory index (C-DII), cardiometabolic risk, and inflammation in adolescents: a cross-sectional study. J Pediatr Endocrinol Metab. (2022) 35:155–62. doi: 10.1515/jpem-2021-0280, 34529909

[92] Petermann-Rocha F Wirth MD Boonpor J Parra-Soto S Zhou Z Mathers JC . Associations between an inflammatory diet index and severe non-alcoholic fatty liver disease: a prospective study of 171,544 UK biobank participants. BMC Med. (2023) 21:123. doi: 10.1186/s12916-023-02793-y, 37013578 PMC10071692

[93] Yan J Zhou J Ding Y Tu C. Dietary inflammatory index is associated with metabolic dysfunction-associated fatty liver disease among United States adults. Front Nutr. (2024) 11:1340453. doi: 10.3389/fnut.2024.1340453, 38559780 PMC10978608

[94] Zhang Y Huang Y Gan X Yang S Zhang Y Wu Y . Pro-inflammatory and pro-oxidant diets and CKD risk across cardiovascular-kidney-metabolic syndrome stages: a multi-omics mediation analysis. Food Funct. (2026) 17:449–60. doi: 10.1039/D5FO03952E, 41379070

[95] Guo C Lin Y Wu S Li H Wu M Wang F. Association of the dietary inflammation index (DII) with the prevalence of chronic kidney disease in patients with type-2 diabetes mellitus. Ren Fail. (2023) 45:2277828. doi: 10.1080/0886022X.2023.2277828, 37994461 PMC11011236

[96] Wirth MD Burch J Shivappa N Violanti JM Burchfiel CM Fekedulegn D . Association of a dietary inflammatory index with inflammatory indices and metabolic syndrome among police officers. J Occup Environ Med. (2014) 56:986–9. doi: 10.1097/JOM.0000000000000213, 25046320 PMC4156884

[97] Christodoulou A Christophi CA Sotos-Prieto M Moffatt S Zhao L Kales SN . The dietary inflammatory index and cardiometabolic parameters in US firefighters. Front Nutr. (2024) 11:1382306. doi: 10.3389/fnut.2024.1382306, 38938668 PMC11208711

[98] Li X Liu L Li X Yang L Men L. Dietary inflammatory index and mortality in middle-aged and elderly patients with metabolic syndrome. Diabetol Metab Syndr. (2025) 17:245. doi: 10.1186/s13098-025-01818-1, 40605106 PMC12220485

[99] Ma Q Zhang Y Zhang D Liu C Zhu W Wang G . The relationship between dietary inflammatory index and all-cause and cardiovascular disease-related mortality in adults with metabolic syndrome: a cohort study of NHANES. Front Endocrinol. (2024) 15:1417840. doi: 10.3389/fendo.2024.1417840 PMC11757130 39866739

[100] Kahn SE Cooper ME Del Prato S. Pathophysiology and treatment of type 2 diabetes: perspectives on the past, present, and future. Lancet. (2014) 383:1068–83. doi: 10.1016/S0140-6736(13)62154-6, 24315620 PMC4226760

[101] Knowler WC Barrett-Connor E Fowler SE Hamman RF Lachin JM Walker EA . Reduction in the incidence of type 2 diabetes with lifestyle intervention or metformin. N Engl J Med. (2002) 346:393–403. doi: 10.1056/NEJMoa012512, 11832527 PMC1370926

[102] Weyer C Bogardus C Mott DM Pratley RE. The natural history of insulin secretory dysfunction and insulin resistance in the pathogenesis of type 2 diabetes mellitus. J Clin Invest. (1999) 104:787–94. doi: 10.1172/JCI7231, 10491414 PMC408438

[103] Mo T Wei M Fu J. Dietary inflammatory index and type 2 diabetes in US women: a cross-sectional analysis of the National Health and nutrition examination survey, 2007-2018. Front Nutr. (2024) 11:1455521. doi: 10.3389/fnut.2024.1455521, 39206319 PMC11351284

[104] Shu Y Wu X Wang J Ma X Li H Xiang Y. Associations of dietary inflammatory index with prediabetes and insulin resistance. Front Endocrinol (Lausanne). (2022) 13:820932. doi: 10.3389/fendo.2022.820932, 35250879 PMC8892213

[105] Jam SA Rezaeian S Najafi F Hamzeh B Shakiba E Moradinazar M . Association of a pro-inflammatory diet with type 2 diabetes and hypertension: results from the Ravansar non-communicable diseases cohort study. Arch Public Health. (2022) 80:102. doi: 10.1186/s13690-022-00839-w, 35361279 PMC8969325

[106] Namazi N Anjom-Shoae J Najafi F Ayati MH Darbandi M Pasdar Y. Pro-inflammatory diet, cardio-metabolic risk factors and risk of type 2 diabetes: a cross-sectional analysis using data from RaNCD cohort study. BMC Cardiovasc Disord. (2023) 23:5. doi: 10.1186/s12872-022-03023-8, 36611151 PMC9825034

[107] Fu W Pei H Shivappa N Hebert JR Luo T Tian T . Association between dietary inflammatory index and type 2 diabetes mellitus in Xinjiang Uyghur autonomous region. China PeerJ. (2021) 9:e11159. doi: 10.7717/peerj.11159, 34316387 PMC8288110

[108] Moslehi N Ehsani B Mirmiran P Shivappa N Tohidi M Hébert JR . Inflammatory properties of diet and glucose-insulin homeostasis in a cohort of Iranian adults. Nutrients. (2016) 8:735. doi: 10.3390/nu8110735, 27869717 PMC5133119

[109] Shen W Cai L Wang B Li J Sun Y Chen Y . Associations of a proinflammatory diet, habitual salt intake, and the onset of type 2 diabetes: a prospective cohort study from the UK biobank. Diabetes Obes Metab. (2024) 26:2119–27. doi: 10.1111/dom.15517, 38409502

[110] Zheng G Cai M Liu H Li R Qian Z Howard SW . Dietary diversity and inflammatory diet associated with all-cause mortality and incidence and mortality of type 2 diabetes: two prospective cohort studies. Nutrients. (2023) 15:120. doi: 10.3390/nu15092120, 37432291 PMC10180882

[111] Wu M Li S Lv Y Liu K Wang Y Cui Z . Associations between the inflammatory potential of diets with adherence to plant-based dietary patterns and the risk of new-onset cardiometabolic diseases in Chinese adults: findings from a nation-wide prospective cohort study. Food Funct. (2023) 14:9018–34. doi: 10.1039/D3FO02579A, 37740363

[112] Jin Q Shi N Aroke D Lee DH Joseph JJ Donneyong M . Insulinemic and inflammatory dietary patterns show enhanced predictive potential for type 2 diabetes risk in postmenopausal women. Diabetes Care. (2021) 44:707–14. doi: 10.2337/dc20-2216, 33419931 PMC7896263

[113] Pang T Alman AC Gray HL Basu A Shi L Snell-Bergeon JK. Empirical dietary inflammatory pattern and metabolic syndrome: prospective association in participants with and without type 1 diabetes mellitus in the coronary artery calcification in type 1 diabetes (CACTI) study. Nutr Res. (2021) 94:1–9. doi: 10.1016/j.nutres.2021.08.001, 34571214 PMC8542609

[114] Sarsangi P Mohammadi M Nadjarzadeh A Salehi-Abargouei A Esmaillzadeh A Mirzaei M. Dietary glycaemic index and insulin index in association with incident type 2 diabetes mellitus in adults. Br J Nutr. (2025) 133:532–43. doi: 10.1017/S0007114524002307, 39927490

[115] Vajdi M Ardekani AM Nikniaz Z Hosseini B Farhangi MA. Dietary insulin index and load and cardiometabolic risk factors among people with obesity: a cross-sectional study. BMC Endocr Disord. (2023) 23:117. doi: 10.1186/s12902-023-01377-4, 37226148 PMC10207709

[116] Liang Y Hu L Shangguan F Tan E Shen J Dai A . Adiposity indices mediate the association between dietary inflammatory index and type 2 diabetes risk in women with prior gestational diabetes mellitus. Diabetol Metab Syndr. (2025) 17:275. doi: 10.1186/s13098-025-01826-1, 40671107 PMC12269103

[117] Xu Y Yao Z Lin J Wei N Yao L. Dietary inflammatory index as a predictor of prediabetes in women with previous gestational diabetes mellitus. Diabetol Metab Syndr. (2024) 16:265. doi: 10.1186/s13098-024-01486-7, 39506813 PMC11542452

[118] Tárnok A Marosvölgyi T Szabó É Györei E Decsi T. Low n-3 long-chain polyunsaturated fatty acids in newly diagnosed celiac disease in children with preexisting type 1 diabetes mellitus. J Pediatr Gastroenterol Nutr. (2015) 60:255–8. doi: 10.1097/MPG.0000000000000561, 25207475

[119] Jiang W Tang Y Yang R Long Y Sun C Han T . Maternal smoking, nutritional factors at different life stage, and the risk of incident type 2 diabetes: a prospective study of the UK biobank. BMC Med. (2024) 22:50. doi: 10.1186/s12916-024-03256-8, 38302923 PMC10835913

[120] Szabo E Marosvolgyi T Decsi T. "Fatty acid supply in pregnant women with type 1 diabetes mellitus". In: Liu C-P, editor. Type 1 Diabetes - Complications, Pathogenesis, and Alternative Treatments. London: IntechOpen (2011)

[121] Alanazi SM Alabonassir OA Almasabi SHA Alhazmi K Alotni MA Mirdad DSA . The association between dietary inflammatory index and diabetic foot ulcers in type 2 diabetes: a case-control study. Front Nutr. (2025) 12:1683264. doi: 10.3389/fnut.2025.1683264, 41170355 PMC12568365

[122] Soltanieh S Salavatizadeh M Poustchi H Yari Z Mansour A Khamseh ME . The association of dietary inflammatory index (DII) and central obesity with non-alcoholic fatty liver disease (NAFLD) in people with diabetes (T2DM). Heliyon. (2023) 9:e13983. doi: 10.1016/j.heliyon.2023.e13983, 36915483 PMC10006473

[123] Xiang W Cheng S Peng Y Jin Q Yang J. DII modulates the relationship between SVD3 and NAFLD prevalence, rather than liver fibrosis severity, in hospitalized T2DM population. Sci Rep. (2024) 14:25567. doi: 10.1038/s41598-024-76560-1, 39462138 PMC11513078

[124] Barta SB Bozkus R Simsek H Kosal B Ucar A. Dietary inflammatory index as a modifiable risk factor for sarcopenia in adults with type 2 diabetes: a cross-sectional study. Nutr Res. (2025) 140:24–33. doi: 10.1016/j.nutres.2025.05.007, 40578013

[125] Pu S Xu Y Tong X Zhang Y Sun X Gao X. Correlation of dietary inflammation index and dietary pattern with mild cognitive impairment in patients with type 2 diabetes. Endocrinol Diabetes Nutr. (2024) 71:152–62. doi: 10.1016/j.endien.2024.01.008, 38735677

[126] Wang B Wang Z Zeng X Fan Y Tang S Yang X . Body mass index as a mediator of the relationship between dietary inflammatory index and depression in diabetes: evidence from the National Health and nutrition examination survey. Medicine (Baltimore). (2025) 104:e44672. doi: 10.1097/MD.0000000000044672, 40988250 PMC12459479

[127] Song W Feng Y Gong Z Tian C. The association between dietary inflammatory index and cognitive performance in older adults aged 60 years and older. Front Nutr. (2022) 9:748000. doi: 10.3389/fnut.2022.748000, 35495906 PMC9039302

[128] Zhang Y Peng Y Deng W Xiang Q Zhang W Liu M. Association between dietary inflammatory index and cognitive impairment among American elderly: a cross-sectional study. Front Aging Neurosci. (2024) 16:1371873. doi: 10.3389/fnagi.2024.1371873, 38550747 PMC10976944

[129] Sun M Wang L Guo Y Yan S Li J Wang X . The association among inflammatory diet, glycohemoglobin, and cognitive function impairment in the elderly: based on the NHANES 2011-2014. J Alzheimer's Dis. (2022) 87:1713–23. doi: 10.3233/JAD-215688, 35491786

[130] Huang L Wang Z Yan S Wang Q Wang L Ye R . Associations of composite dietary antioxidant index and dietary inflammation index with cognitive dysfunction in older Chinese adults: results from China health and nutrition survey in 2018. Nutrients. (2025) 17:412. doi: 10.3390/nu17213412, 41228485 PMC12609353

[131] Huang H Li J Shen J Zhao T Xiao R Ma W. Dietary inflammatory index and cognitive function: findings from a cross-sectional study in obese Chinese township population from 45 to 75 years. J Inflamm Res. (2024) 17:2365–82. doi: 10.2147/JIR.S447300, 38651005 PMC11034566

[132] Chen L Liu J Li X Hou Z Wei Y Chen M . Energy-adjusted dietary inflammatory index and cognitive function in Chinese older adults: a population-based cross-sectional study. Nutr Neurosci. (2024) 27:978–88. doi: 10.1080/1028415X.2023.2285537, 37992128

[133] Liu K Zhong Y Cen P Dong L Chen Y Hu D . Sex differences in the association between energy-adjusted dietary inflammatory index and cognitive function in older adults. Alzheimers Dement. (2025) 21:e70908. doi: 10.1002/alz.70908, 41243144 PMC12620079

[134] Shi Y Lin F Li Y Wang Y Chen X Meng F . Association of pro-inflammatory diet with increased risk of all-cause dementia and Alzheimer's dementia: a prospective study of 166,377 UK biobank participants. BMC Med. (2023) 21:266. doi: 10.1186/s12916-023-02940-5, 37480061 PMC10362711

[135] Peng M Yuan S Lu D Ling Y Huang X Lyu J . Dietary inflammatory index, genetic susceptibility and risk of incident dementia: a prospective cohort study from UK biobank. J Neurol. (2024) 271:1286–96. doi: 10.1007/s00415-023-12065-7, 37985486

[136] van Lent DM Mesa HG Short MI Gonzales MM Aparicio HJ Salinas J . Association between dietary inflammatory index score and incident dementia. Alzheimers Dement. (2025) 21:e14390. doi: 10.1002/alz.14390, 39641390 PMC11772702

[137] Charisis S Ntanasi E Yannakoulia M Anastasiou CA Kosmidis MH Dardiotis E . Diet inflammatory index and dementia incidence: a population-based study. Neurology. (2021) 97:e2381–91. doi: 10.1212/WNL.0000000000012973, 34759053 PMC8673721

[138] Liao R Yang J Huang X Xu Y Ji Q Liu Q . Dietary inflammatory index and Alzheimer's disease mortality in a prospective cohort. Exp Gerontol. (2025) 206:112770. doi: 10.1016/j.exger.2025.112770, 40318706

[139] Hsu CC Wang SI Yu S Lin ES Wei JC. Adherence to an anti-inflammatory diet is associated with lower Alzheimer's disease mortality: a modifiable risk factor in a national cohort. J Prev Alzheimers Dis. (2025) 12:100221. doi: 10.1016/j.tjpad.2025.100221, 40517083 PMC12413709

[140] Fang B Wang Z Nan G. Dietary inflammatory potential and the risk of cognitive impairment: a meta-analysis of prospective cohort studies. J Nutr Health Aging. (2025) 29:100428. doi: 10.1016/j.jnha.2024.100428, 39689376 PMC12180060

[141] Melo Van Lent D Gokingco H Short MI Yuan C Jacques PF Romero JR . Higher dietary inflammatory index scores are associated with brain MRI markers of brain aging: results from the Framingham heart study offspring cohort. Alzheimers Dement. (2023) 19:621–31. doi: 10.1002/alz.12685, 35522830 PMC9637238

[142] Dove A Dunk MM Wang J Guo J Whitmer RA Xu W. Anti-inflammatory diet and dementia in older adults with cardiometabolic diseases. JAMA Netw Open. (2024) 7:e2427125. doi: 10.1001/jamanetworkopen.2024.27125, 39133488 PMC11320167

[143] Dunk MM Huang H Wang J Dove A Sakakibara S Guo J . The association between a pro-inflammatory diet and brain age in middle-aged and older adults. Eur J Epidemiol. (2025) 41:39–50. doi: 10.1007/s10654-025-01318-6 41144113 PMC12881069

[144] van Lent D.M. Jacques P.F. Charisis S.M. Mesa H.G. Satizabal C. Yuan C. ., Dietary inflammatory index scores and cognitive aging: results from the Framingham heart study offspring cohort. medRxiv [Preprint] (2024). doi: 10.1101/2024.11.27.24318084

[145] Melo van Lent D Samieri C Grodstein F Seshadri S. Empirical dietary inflammatory pattern scores are not associated with worse cognitive performance in the nurses' health study. J Nutr. (2022) 152:2526–33. doi: 10.1093/jn/nxac157, 36774118 PMC9644169

[146] Dai J Chan DKY Chan RO Hirani V Xu YH Braidy N. The association between dietary patterns, plasma lipid profiles, and inflammatory potential in a vascular dementia cohort. Aging Med. (2023) 6:155–62. doi: 10.1002/agm2.12249, 37287668 PMC10242272

[147] Wang X Li T Li H Li D Wang X Zhao A . Association of Dietary Inflammatory Potential with blood inflammation: the prospective markers on mild cognitive impairment. Nutrients. (2022) 14:417. doi: 10.3390/nu14122417 35745147 PMC9229190

[148] Liu Q Li Z Huang L Zhou D Fu J Duan H . Telomere and mitochondria mediated the association between dietary inflammatory index and mild cognitive impairment: a prospective cohort study. Immun Ageing. (2023) 20:1. doi: 10.1186/s12979-022-00326-4, 36604719 PMC9813461

[149] Tao L Yang Q Zeng X Yu H Mu J Yan Z . Association between food inflammation scores of individuals and sarcopenia in U.S. adults: a cross-sectional study from NHANES 2011-2018. Exp Gerontol. (2026) 213:113004. doi: 10.1016/j.exger.2025.113004, 41397661

[150] Yang Q Cai X Xiao Z. Higher food inflammation index is linearly associated with higher risk of MASLD: a cross-sectional study based on the NHANES (1999-2020). Food Sci Nutr. (2025) 13:e70865. doi: 10.1002/fsn3.70865, 40905021 PMC12402601

[151] Fan YC Chou CC Bintoro BS Chien KL Bai CH. High sensitivity C-reactive protein and glycated hemoglobin levels as dominant predictors of all-cause dementia: a nationwide population-based cohort study. Immun Ageing. (2022) 19:10. doi: 10.1186/s12979-022-00265-0, 35172860 PMC8849019

[152] Cho S Ok Kim C Cha BS Kim E Mo Nam C Kim MG . The effects of long-term cumulative HbA1c exposure on the development and onset time of dementia in the patients with type 2 diabetes mellitus: hospital based retrospective study (2005-2021). Diabetes Res Clin Pract. (2023) 201:110721. doi: 10.1016/j.diabres.2023.110721, 37196708

[153] Navratilova HF Whetton AD Geifman N. Integrating food preference profiling, behavior change strategies, and machine learning for cardiovascular disease prevention in a personalized nutrition digital health intervention: conceptual pipeline development and proof-of-principle study. J Med Internet Res. (2025) 27:e75106. doi: 10.2196/75106, 40808315 PMC12346185

[154] Guo D Yang D Zhang H Song J Wang P Zhu Q . DeepSeek-R1 incentivizes reasoning in LLMs through reinforcement learning. Nature. (2025) 645:633–8. doi: 10.1038/s41586-025-09422-z, 40962978 PMC12443585

[155] Amini Y Saif N Greer C Hristov H Isaacson R. The role of nutrition in individualized Alzheimer's risk reduction. Curr Nutr Rep. (2020) 9:55–63. doi: 10.1007/s13668-020-00311-7, 32277428

[156] Uribarri J Woodruff S Goodman S Cai W Chen X Pyzik R . Advanced glycation end products in foods and a practical guide to their reduction in the diet. J Am Diet Assoc. (2010) 110:911–16.e12. doi: 10.1016/j.jada.2010.03.018, 20497781 PMC3704564

[157] Marco ML Heeney D Binda S Cifelli CJ Cotter PD Foligné B . Health benefits of fermented foods: microbiota and beyond. Curr Opin Biotechnol. (2017) 44:94–102. doi: 10.1016/j.copbio.2016.11.010, 27998788

[158] Khan S Wirth MD Ortaglia A Alvarado CR Shivappa N Hurley TG . Design, development and construct validation of the children's dietary inflammatory index. Nutrients. (2018) 10:993. doi: 10.3390/nu10080993, 30061487 PMC6115957

[159] Shivappa N Wirth MD Murphy EA Hurley TG Hébert JR. Association between the dietary inflammatory index (DII) and urinary enterolignans and C-reactive protein from the National Health and nutrition examination Survey-2003-2008. Eur J Nutr. (2019) 58:797–805. doi: 10.1007/s00394-018-1690-5, 29675557

[160] Du M Deng K Yin J Wu C Hu S Guo L . Association between chewing capacity and mortality risk: the role of diet and ageing. J Clin Periodontol. (2025) 52:695–706. doi: 10.1111/jcpe.14122, 39800933

[161] Fauzi A Thoe ES Quan TY Yin ACY. Insights from insulin resistance pathways: therapeutic approaches against Alzheimer associated diabetes mellitus. J Diabetes Complicat. (2023) 37:108629. doi: 10.1016/j.jdiacomp.2023.108629, 37866274

[162] Parolini C. Marine n-3 polyunsaturated fatty acids: efficacy on inflammatory-based disorders. Life Sci. (2020) 263:118591. doi: 10.1016/j.lfs.2020.118591, 33069735

[163] Zhang L Virgous C Si H. Synergistic anti-inflammatory effects and mechanisms of combined phytochemicals. J Nutr Biochem. (2019) 69:19–30. doi: 10.1016/j.jnutbio.2019.03.009 31048206

[164] Paula VG Vesentini G Sinzato YK Moraes-Souza RQ Volpato GT Damasceno DC. Intergenerational high-fat diet impairs ovarian follicular development in rodents: a systematic review and meta-analysis. Nutr Rev. (2022) 80:889–903. doi: 10.1093/nutrit/nuab049, 34459492

[165] Wang S Chen Z Cai Y Wu X-L Wang S Tang Z . Application of COFs in capture/conversion of CO2 and elimination of organic/inorganic pollutants. Environ Funct Mater. (2023) 2:76–92. doi: 10.1016/j.efmat.2023.03.001

[166] Zhu G Zhao J Zhang H Wang G Chen W. Gut microbiota and its metabolites: bridge of dietary nutrients and Alzheimer's disease. Adv Nutr. (2023) 14:819–39. doi: 10.1016/j.advnut.2023.04.005, 37075947 PMC10334159

[167] Banaszak M Górna I Woźniak D Przysławski J Drzymała-Czyż S. The impact of curcumin, resveratrol, and cinnamon on modulating oxidative stress and antioxidant activity in type 2 diabetes: moving beyond an anti-hyperglycaemic evaluation. Antioxidants. (2024) 13:510. doi: 10.3390/antiox13050510 38790615 PMC11117755

