# Supplementary material for: From nutrient-based to food-based assessment: the evolution of inflammatory indices and their significance for metabolic syndrome and type 3 diabetes mellitus
Source: Front Nutr. 2026 May 14;13:1818219. doi: 10.3389/fnut.2026.1818219 (PMC13215941; doi:10.3389/fnut.2026.1818219)
Supplement: Supplementary file 1 [file Table_1.DOCX]

Supplementary Material

The following supplementary materials provide additional details supporting the main text of the study.

**Supplementary Information S1.** Calculation of FISI34, FISI26-USDA, FISI26-CHINA and DII

**Supplementary Table S1.** The total inflammation score (TIS) and nutrient recommended values (NRVs) of nutrient components for Food Inflammation Score of Individuals (FISI) Calculations

**Supplementary Table S2.** Food Parameters, Inflammatory Effect Scores, and Global Intake Values for Dietary Inflammatory Index (DII)

**Supplementary Information S1.** Methodological Framework for Calculating the Dietary Inflammatory Index (DII) and Food Inflammation Score of Individuals (FISI)

**Overview**

This document provides the standardized methodological framework for calculating the Dietary Inflammatory Index (DII) and the Food Inflammation Score of Individuals (FISI). These indices are utilized in nutritional epidemiology to quantify the inflammatory potential of an individual’s diet, transitioning from nutrient-centric assessments to holistic food-matrix-based evaluations.

**1.Food Inflammation Score of Individuals (FISI) Framework**

The FISI is a food-based inflammatory assessment tool derived from the Food Inflammation Index (FII) framework. It evaluates the inflammatory potential of whole foods by integrating the physiological impact of their constituent nutrients. Depending on the granularity of available Food Composition Tables (FCTs), researchers may utilize different FISI variants.

**FISI34 (Expanded Nutrient Profile)**

**FISI34** is a refined version of the original FISI39 framework, optimized for databases with specific bioactive reporting constraints. It encompasses 34 dietary components, with Nutrient Reference Values (NRVs) typically based on the U.S. Dietary Guidelines for Americans (DGA) for adults (e.g., individuals aged 31–50 at a 2200 kcal level).

Components included: Protein, Total fat, Carbohydrate, Energy, Alcohol, Caffeine, Fiber, Iron, Magnesium, Zinc, Selenium, Vitamin A (RAE), Alpha-carotene, Vitamin E (alpha-tocopherol), Vitamin D, Vitamin C, Thiamin, Riboflavin, Niacin, Vitamin B6, Vitamin B12, Folic acid, Added Vitamin E, Added Vitamin B12, Cholesterol, Saturated fatty acids, MUFA, PUFA, and specific fatty acid fractions (PUFA 18:2, 18:3, 20:4, 22:6 n-3, 20:5 n-3, 22:5 n-3).

**FISI26 Variants (Standard Nutrient Profile)**

**FISI26** is designed for broader clinical application where data on specific bioactives or added vitamins may be limited. [1]

**FISI26-USDA**: Utilizes 26 major dietary components with NRVs based on the DGA 2020-2025.

**FISI26-CHINA**: Utilizes the same 26 components but adapts the NRVs according to the Chinese Dietary Reference Intakes (DRIs) for specific demographics (e.g., males aged 50).

**FISI Scoring Algorithm:**

The individual FISI score is calculated as the sum of the inflammatory contributions of all included nutrients:

The FISI calculation follows the formula:

$FISI=\sum\frac{{TIS}_{i}\times{Intake}_{i}}{{NRV}_{i}}$ (1)

(1) Where: ${Intake}_{i}$ is the individual's average daily intake of the i-th nutrient, ${TIS}_{i}$ is the total inflammatory tendency score for the corresponding nutrient, and ${NRV}_{i}$ is the reference nutrient intake for that nutrient.

**2. Dietary Inflammatory Index (DII) Calculation Methodology**

The DII calculation follows the literature-based standardized methodology developed to assess the overall inflammatory load of the diet across 45 parameters (28 of which are most commonly utilized in clinical research). [2]

Standardized Components (28 items):
Carbohydrates, Protein, Alcohol, Fiber, Cholesterol, Total fat, Saturated fat, MUFAs, PUFAs, n-3 fatty acids, n-6 fatty acids, Vitamins (A, B1, B2, B3, B6, B12, C, D, E), Minerals (Fe, Mg, Zn, Se), Folic acid, Beta-carotene, Caffeine, and Total Energy.

Scoring Logic:

1. Z-score Calculation: For each component, a z-score is calculated by subtracting the global daily mean and dividing by the global standard deviation (from the world referent database).
2. Percentile Conversion: Z-scores are converted to centered percentiles to mitigate the influence of right-skewed data.
3. Weighting: Each centered percentile is multiplied by the component’s specific inflammatory effect score (derived from approximately 1,943 research articles).
4. Aggregation: The weighted scores are summed to generate the individual’s overall DII score.

**References**

1. YX Y: **Society CN,Dietary Reference Intakes for China**. Beijing: People's Medical Publishing House; 2023.

2. Shivappa N, Steck SE, Hurley TG, Hussey JR, Hébert JR: **Designing and developing a literature-derived, population-based dietary inflammatory index**. *Public Health Nutr* 2014, **17**(8):1689–1696.

**Supplementary Table S1.** Nutrient Components, Total Inflammation Scores (TIS), and Nutrient Recommended Values (NRVs) for Food Inflammation Score of Individuals (FISI) Calculations

| **Food Parameter** | **Unit** | **TIS** | **NRV (DGA)** | **NRV (China)** | **FISI34** | **FISI26** | **Inclusion Notes** |
| --- | --- | --- | --- | --- | --- | --- | --- |
| Macronutrients & Energy |  |  |  |  |  |  |  |
| Energy | kcal | 0.18 | 2200 | 1950 | Yes | Yes | Core parameter |
| Protein | g | 0.021 | 56 | 65 | Yes | Yes | Core parameter |
| Total fat | g | 0.298 | 77 | 54.2 | Yes | Yes | Core parameter |
| Carbohydrate | g | 0.097 | 130 | 120 | Yes | Yes | Core parameter |
| Fiber total dietary | g | -0.663 | 31 | 30 | Yes | Yes | Core parameter |
| Vitamins |  |  |  |  |  |  |  |
| Vitamin A (RAE) | ug | -0.401 | 900 | 750 | Yes | Yes |  |
| Beta-Carotene | ug | -0.584 | 3718 | 3718 | Yes | Yes |  |
| Vitamin C | mg | -0.424 | 90 | 100 | Yes | Yes |  |
| Vitamin D | ug | -0.446 | 15 | 10 | Yes | No | Excluded in FISI-26 (FCT limit) |
| Vitamin E | mg | -0.419 | 15 | 14 | Yes | Yes | Alpha-tocopherol |
| Thiamin (B1) | mg | -0.098 | 1.2 | 1.4 | Yes | Yes |  |
| Riboflavin (B2) | mg | -0.068 | 1.3 | 1.4 | Yes | Yes |  |
| Niacin (B3) | mg | -0.246 | 16 | 15 | Yes | Yes |  |
| Vitamin B6 | mg | -0.365 | 1.3 | 1.4 | Yes | No | Excluded in FISI-26 (FCT limit) |
| Folic acid | ug | -0.19 | 400 | 400 | Yes | No | Excluded in FISI-26 (FCT limit) |
| Vitamin B12 | ug | 0.106 | 2.4 | 2.4 | Yes | No | Excluded in FISI-26 (FCT limit) |
| **Minerals** |  |  |  |  |  |  |  |
| Magnesium Mg | mg | -0.484 | 420 | 320 | Yes | Yes |  |
| Iron Fe | mg | 0.032 | 8 | 12 | Yes | Yes |  |
| Zinc Zn | mg | -0.313 | 11 | 12 | Yes | Yes |  |
| Selenium Se | ug | -0.191 | 67 | 60 | Yes | Yes |  |
| **Lipid Fractions** |  |  |  |  |  |  |  |
| Cholesterol | mg | 0.11 | 279.4 | 279.4 | Yes | Yes |  |
| SFA (Saturated Fat) | g | 0.373 | 22 | 21.7 | Yes | Yes |  |
| MUFA | g | -0.009 | 27 | 27 | Yes | Yes |  |
| PUFA (Total) | g | -0.337 | 13.9 | 15.2 | Yes | Yes |  |
| n-3 Fatty acids | g | -0.436 | 1.06 | 2.71 | Yes | Yes | DHA/EPA/DPA |
| n-6 Fatty acids | g | -0.159 | 10.8 | 12.5 | Yes | Yes |  |
| Trans fat | g | 0.229 | 2.2 | - | No | No | Limited FCT data |
| Bioactives & Flavonoids |  |  |  |  |  |  | |
| Flavan-3-ols | mg | -0.415 | 95.8 | - | Yes | No | Flavonoid subgroup |
| Flavanones | mg | -0.25 | 11.7 | - | Yes | No | Flavonoid subgroup |
| Flavones | mg | -0.616 | 1.55 | - | Yes | No | Flavonoid subgroup |
| Flavonols | mg | -0.467 | 17.7 | - | Yes | No | Flavonoid subgroup |
| Isoflavones | mg | -0.593 | 1.2 | - | Yes | No | Flavonoid subgroup |
| Anthocyanidins | mg | -0.131 | 18.1 | - | No | No | Limited FCT data |
| Caffeine | mg | -0.11 | 8.05 | - | Yes | No |  |
| Alcohol | g | -0.278 | 5.15 | - | Yes | No |  |
| Eugenol | mg | -0.14 | 0.01 | - | No | No | Limited FCT data |
| Whole Food Parameters |  |  |  |  |  |  | |
| Garlic | g | -0.412 | - | - | No | No | Re-classified as Food Item |
| Ginger | g | -0.453 | - | - | No | No | Re-classified as Food Item |
| Onion | g | -0.301 | - | - | No | No | Re-classified as Food Item |
| Green/Black Tea | g | -0.536 | - | - | No | No | Re-classified as Food Item |
| Pepper | g | -0.131 | - | - | No | No | Re-classified as Food Item |
| Saffron | g | -0.14 | - | - | No | No | Re-classified as Food Item |
| Turmeric | mg | -0.785 | - | - | No | No | Re-classified as Food Item |
| Thyme/Oregano | mg | -0.102 | - | - | No | No | Re-classified as Food Item |
| Rosemary | mg | -0.013 | - | - | No | No | Re-classified as Food Item |

Notes:

TIS (Total Inflammation Score): The "Overall Inflammatory Effect Score" from Shivappa et al. (2014).

NRV (DGA): Recommended values based on Dietary Guidelines for Americans 2020-2025 (2200 kcal level).

NRV (China): Values from Chinese Dietary Reference Intakes (DRIs) 2023 for adult males (18-50y).

FISI Inclusion: "Yes" indicates the parameter is included in the individual Food Inflammation Score calculation based on FCT data.

Flavonoid Standards: Values represent mean intakes from referent populations used as placeholders for NRVs.

Exclusion Logic: Parameters listed as "No" under FISI (e.g., Garlic, Onion) were excluded from the nutrient-based calculation because they are defined as whole foods or specific ingredients rather than isolated nutrients in Food Composition Tables (FCT). Their inflammatory effects are captured by the FII score assigned to the food items themselves.

Supplementary Table S2. Literature-Derived Inflammatory Weights and Global Referent Database for the Dietary Inflammatory Index (DII)

Description: This table presents the complete set of 45 food parameters used in the DII. It includes the robustness of the literature (number of articles), the inflammatory effect scores, and the global referent values (Mean and SD) used for Z-score standardization.

| **Food Parameter** | **Raw Inflammatory Effect Score*** | **Overall Inflammatory Effect Score†** | **Global Daily Mean Intake‡ (units/d)** | **sd‡** |
| --- | --- | --- | --- | --- |
| Macronutrients & Energy |  |  |  |  |
| Energy (kcal) | 0.18 | 0.18 | 2056 | 338 |
| Protein (g) | 0.049 | 0.021 | 79.4 | 13.9 |
| Total fat (g) | 0.298 | 0.298 | 71.4 | 19.4 |
| Carbohydrate (g) | 0.109 | 0.097 | 272.2 | 40 |
| Fibre (g) | -0.663 | -0.663 | 18.8 | 4.9 |
| Vitamins |  |  |  |  |
| Vitamin A (RE) | -0.401 | -0.401 | 983.9 | 518.6 |
| Beta-Carotene (ug) | -0.584 | -0.584 | 3718 | 1720 |
| Vitamin C (mg) | -0.424 | -0.424 | 118.2 | 43.46 |
| Vitamin D (ug) | -0.446 | -0.446 | 6.26 | 2.21 |
| Vitamin E (mg) | -0.419 | -0.419 | 8.73 | 1.49 |
| Thiamin (mg) | -0.354 | -0.098 | 1.7 | 0.66 |
| Riboflavin (mg) | -0.727 | -0.068 | 1.7 | 0.79 |
| Niacin (mg) | -1 | -0.246 | 25.9 | 11.77 |
| Vitamin B6 (mg) | -0.379 | -0.365 | 1.47 | 0.74 |
| Folic acid (ug) | -0.207 | -0.19 | 273 | 70.7 |
| Vitamin B12 (ug) | 0.205 | 0.106 | 5.15 | 2.7 |
| Minerals |  |  |  |  |
| Magnesium (mg) | -0.484 | -0.484 | 310.1 | 139.4 |
| Iron (mg) | 0.032 | 0.032 | 13.35 | 3.71 |
| Zinc (mg) | -0.313 | -0.313 | 9.84 | 2.19 |
| Selenium Se (ug) | -0.191 | -0.191 | 67 | 25.1 |
| Lipids & Fatty Acids |  |  |  |  |
| Cholesterol (mg) | 0.347 | 0.11 | 279.4 | 51.2 |
| Saturated fat (g) | 0.429 | 0.373 | 28.6 | 8 |
| MUFA (g) | -0.019 | -0.009 | 27 | 6.1 |
| PUFA (g) | -0.337 | -0.337 | 13.88 | 3.76 |
| n-3 Fatty acids (g) | -0.436 | -0.436 | 1.06 | 1.06 |
| n-6 Fatty acids (g) | -0.159 | -0.159 | 10.8 | 7.5 |
| Trans fat (g) | 0.432 | 0.229 | 3.15 | 3.75 |
| Bioactive Compounds |  |  |  |  |
| Alcohol (g) | -0.278 | -0.278 | 13.98 | 3.72 |
| Caffeine (g) | -0.124 | -0.11 | 8.05 | 6.67 |
| Anthocyanidins (mg) | -0.449 | -0.131 | 18.05 | 21.14 |
| Flavan-3-ol (mg) | -0.415 | -0.415 | 95.8 | 85.9 |
| Flavonones (mg) | -0.908 | -0.25 | 11.7 | 3.82 |
| Flavones (mg) | -0.616 | -0.616 | 1.55 | 0.07 |
| Flavonols (mg) | -0.467 | -0.467 | 17.7 | 6.79 |
| Isoflavones (mg) | -0.593 | -0.593 | 1.2 | 0.2 |
| Whole Foods & Spices |  |  |  |  |
| Garlic (g) | -0.412 | -0.412 | 4.35 | 2.9 |
| Ginger (g) | -0.588 | -0.453 | 59 | 63.2 |
| Onion (g) | -0.49 | -0.301 | 35.9 | 18.4 |
| Green/black tea (g) | -0.536 | -0.536 | 1.69 | 1.53 |
| Pepper (g) | -0.397 | -0.131 | 10 | 7.07 |
| Turmeric (mg) | -0.785 | -0.785 | 533.6 | 754.3 |
| Saffron (g) | -1 | -0.14 | 0.37 | 1.78 |
| Eugenol (mg) | -0.868 | -0.14 | 0.01 | 0.08 |
| Thyme/oregano (mg) | -1 | -0.102 | 0.33 | 0.99 |
| Rosemary (mg) | -0.333 | -0.013 | 1 | 15 |

Note: RE, retinol equivalents; *: The value of Raw Inflammatory Effect Score, overall inflammatory effect score, global daily mean intake (units/d) and sd are derived from references (Shivappa N, Steck SE, Hurley TG, Hussey JR, Hebert JR. Designing and developing a literature-derived, population-based dietary inflammatory index. Public Health Nutr. 2014;17:1689-96.).[2]
